# Improved Adsorption of an *Enterococcus faecalis* Bacteriophage ΦEF24C with a Spontaneous Point Mutation

**DOI:** 10.1371/journal.pone.0026648

**Published:** 2011-10-25

**Authors:** Jumpei Uchiyama, Iyo Takemura, Miho Satoh, Shin-ichiro Kato, Takako Ujihara, Kazue Akechi, Shigenobu Matsuzaki, Masanori Daibata

**Affiliations:** 1 Department of Microbiology and Infection, Faculty of Medicine, Kochi University, Kochi, Japan; 2 Science Research Center, Kochi University, Kochi, Japan; Cairo University, Egypt

## Abstract

Some bacterial strains of the multidrug-resistant Gram-positive bacteria *Enterococcus faecalis* can significantly reduce the efficacy of conventional antimicrobial chemotherapy. Thus, the introduction of bacteriophage (phage) therapy is expected, where a phage is used as a bioagent to destroy bacteria. *E. faecalis* phage ΦEF24C is known to be a good candidate for a therapeutic phage against *E. faecalis*. However, this therapeutic phage still produces nonuniform antimicrobial effects with different bacterial strains of the same species and this might prove detrimental to its therapeutic effects. One solution to this problem is the preparation of mutant phages with higher activity, based on a scientific rationale. This study isolated and analyzed a spontaneous mutant phage, ΦEF24C-P2, which exhibited higher infectivity against various bacterial strains when compared with phage ΦEF24C. First, the improved bactericidal effects of phage ΦEF24C-P2 were attributable to its increased adsorption rate. Moreover, genomic sequence scanning revealed that phage ΦEF24C-P2 had a point mutation in *orf31*. Proteomic analysis showed that ORF31 (mw, 203 kDa) was present in structural components, and immunological analysis using rabbit-derived antibodies showed that it was a component of a long, flexible fine tail fiber extending from the tail end. Finally, phage ΦEF24C-P2 also showed higher bactericidal activity in human blood compared with phage ΦEF24C using the in vitro assay system. In conclusion, the therapeutic effects of phage ΦEF24C-P2 were improved by a point mutation in gene *orf31*, which encoded a tail fiber component.

## Introduction

*Enterococcus* species are opportunistic pathogens. Vancomycin-resistant *Enterococcus* (VRE) species are medically important because they fail to respond to conventional antimicrobial chemotherapy and cause fatal infections in nosocomial settings. An increase in the number of VRE cases has been reported worldwide, and *E. faecalis* is the most frequent clinically isolated species in enterococcal infections [1]–[6]. Although the situation is critical, antimicrobial agents with new modes of action are unlikely to be developed because there is little commercial interest in producing novel antibiotics or in developing novel technology [7], [8]. Therefore, to combat this bacterium, an alternative therapeutic approach to antibiotics must be explored. One of the favored alternative therapies to antibiotics is phage therapy, which uses bacteriophages (bacterial viruses) as selective bioagents to destroy the targeted bacteria.

In addition to its effectiveness against drug-resistant bacteria, phage therapy has many superior characteristics compared with antibiotics, including the phage targeting ability, multiplication at the site of infection, and fewer side effects [9]–[11]. However, phage therapy is not always ideal because, just as different bacterial strains show different levels of antibiotic sensitivity, different strains also vary in their sensitivity to phage [12], [13]. The sensitivity of bacteria to phage can be measured as phage infectivity for a bacterial strain; this can be expressed as the relative efficiency of plating (EOP), which is the ratio of plaque-forming units (pfu) of phage plated on a particular bacterial strain to the pfu of phage plated on a standard strain. EOP variation may influence the therapeutic effects. In some cases, bacterial strain with low EOP may not result in the desired clinical outcome.

Phages with strong lytic activity and a broad host range are generally used in phage therapy. We have previously reported an *E. faecalis* phage ΦEF24C with strong lytic activity and an extremely broad spectrum of hosts. This phage belongs to the family *Myoviridae* and the genus of SPO1-like viruses, which are common member lytic phages that have been applied in phage therapy [14]–[17]. Previous analysis of phage ΦEF24C using in silico, in vitro, and in vivo methods indicated the safety and therapeutic effects of phage ΦEF24C, so it was eligible as a therapeutic phage candidate [16]–[18]. However, the therapeutic effect of phage ΦEF24C varies between different bacterial strains. For example, phage ΦEF24C infected the *E. faecalis* strain EF14 at a rate ca. 32 times higher than for the vancomycin-resistant *E. faecalis* strain VRE2, which required a tenfold higher concentration of phage ΦEF24C to rescue 100% of mice infected with strain VRE2 compared with strain EF14 [18].

In the future, it is expected that highly active phages will be produced by genetic recombination or natural mutation with the support of a sound scientific rationale [9], [19], and so some efforts have recently been initiated [20]–[25]. This demands greater accumulation of knowledge regarding phage biology, particularly study of SPO1-like viruses. To address this goal, the analysis of highly active mutant phages with improved therapeutic efficacy could be used as a shortcut in the development of therapeutic phage technology. In the current study, a spontaneous mutant phage with improved therapeutic efficacy was isolated from the therapeutic candidate phage ΦEF24C. The molecular causes of its enhanced efficacy were explored by comparison with phage ΦEF24C.

## Materials and Methods

### Bacteria and phage culture and plasmids

Thirty-five *E. faecalis* strains are described in Table 1. *Escherichia coli* strains DH5α and BL21 were used for cloning and protein overexpression. *E. faecalis* phage ΦEF24C is described elsewhere [16]–[18]. pUC18 and pCold II plasmids were purchased from Takara Bio (Kyoto, Japan) for the purposes of cloning and protein overexpression, respectively.

**Table 1:** *E. faecalis* strains used in this study and phage infectious activity.

					Efficiency of plating (EOP)		
*E. faecalis* strain	Description^a^	Isolated source	Phage ΦEF24C activity	Phage ΦEF24C-P2 activity	ΦEF24C	ΦEF24C-P2	EOP relative rate (ΦEF24C-P2/ΦEF24C)
ATCC19433	ATCC standard strain		○	○	0.78	0.64	0.82
EF1	Clinical isolate from KUH	Vaginal discharge	○	○	1.40	2.70	1.93
EF2	Clinical isolate from KUH	Vaginal discharge	○	○	0.80	2.00	2.50
EF3	Clinical isolate from KUH	Sputum	○	○	0.50	0.80	1.60
EF4	Clinical isolate from KUH	Pharyngis	–	–			
EF5	Clinical isolate from KUH	Skin	○	○	0.90	2.50	2.78
EF6*	Clinical isolate from KUH	Urine	○	○	0.06	1.50	25.00
EF7	Clinical isolate from KUH	Eye discharge	○	○	1.40	2.50	1.79
EF8	Clinical isolate from KUH	Other	○	○	0.70	2.20	3.14
EF9*	Clinical isolate from KUH	Urine	○	○	0.10	2.60	26.00
EF10	Clinical isolate from KUH	Other	○	○	1.00	2.80	2.80
EF11*	Clinical isolate from KUH	Urine	○	○	0.08	1.70	21.25
EF12*	Clinical isolate from KUH	Urine	○	○	0.06	2.20	36.67
EF13	Clinical isolate from KUH	Pharyngis	–	–			
EF14	Clinical isolate from KUH	Vaginal discharge	○	○	1.00	1.30	1.30
EF15	Clinical isolate from KUH	Pus	–	–			
EF16	Clinical isolate from KUH	Vaginal discharge	○	○	0.80	2.60	3.25
EF17	Clinical isolate from KUH	Vaginal discharge	○	○	0.60	1.60	2.67
EF18	Clinical isolate from KUH	Vaginal discharge	○	○	0.90	1.00	1.11
EF19	Clinical isolate from KUH	Vaginal discharge	○	○	1.40	3.00	2.14
EF20	Clinical isolate from KUH	Pus	○	○	0.20	1.00	5.00
EF21	Clinical isolate from KUH	Pus	○	○	1.00	1.50	1.50
EF22	Clinical isolate from KUH	Mouthwash	○	○	1.20	1.70	1.42
EF23	Clinical isolate from KUH	Sputum	○	○	0.20	1.20	6.00
EF24	Clinical isolate from KUH	Vaginal discharge	○	○	**1.00**	**1.00**	1.00
EF25	Clinical isolate from KUH	Other	○	○	1.00	1.50	1.50
EF26	Clinical isolate from KUH	Vaginal discharge	○	○	0.90	1.10	1.22
EF27	Clinical isolate from KUH	Eye discharge	○	○	0.80	0.50	0.63
EF28	Clinical isolate from KUH	Vaginal discharge	○	○	0.70	1.20	1.71
EF29	Clinical isolate from KUH	Pus	○	○	0.70	1.40	2.00
EF30	Clinical isolate from KUH	Vaginal discharge	–	–			
VRE1	VRE strain	Sputum	○	○	0.22	1.46	6.64
VRE2*	VRE strain	Urine	○	○	0.03	1.61	50.31
VRE3*	VRE strain	Urine	○	○	0.05	1.46	30.42
VRE4*	VRE strain	Rectal swab	○	○	0.02	1.57	65.42

a KUH represents Kochi University Hospital; all VRE strains were isolated from Nagano Hokushin Hospital, Nagano, Japan.

“○” indicates plaque forming, and “–” indicates “lysis from without” or no lysis.

*E. faecalis strain with relative EOP rates greater than 10 (ΦEF24C-P2 EOP/ΦEF24C).

**Measurements were conducted twice and representative data are shown here.**

Tryptic soy broth (TSB) was used as a culture medium for *E. faecalis* and phage ΦEF24C. Luria–Bertani (LB) medium containing 100 µg mL^−1^ ampicillin was used as a culture medium for *Esc. coli* harboring the appropriate plasmid. All bacteria and phage incubations were conducted in a TSB medium at 37°C, unless otherwise stated. Phages were cultured in either liquid media or double-layered agar with the appropriate *E. faecalis* bacterial strain. In the double-layered agar method, 100 µL of bacterial overnight culture was plated with or without 100 µL of phage suspension.

Unless otherwise stated, all culture media and reagents used in this study were purchased from Becton, Dickinson and Co. (Sparks, MD, USA), and Nacalai Tesque Inc. (Kyoto, Japan), respectively.

### Isolation of the *E. faecalis* phage ΦEF24C spontaneous mutant

Phage ΦEF24C was diluted to approximately 1.0×10^4^ pfu mL^−1^, and 100 µL of the diluted phage was plated with the *E. faecalis* strain VRE2. A large clear plaque was purified at least three times.

Plaque sizes were compared to examine the difference between wild-type and mutant phages. After incubation of wild-type or mutant phage on the double-layered agar with *E. faecalis* strain VRE2, the bacterial lawn was stained with 2% 2,3,5,-triphenyltetrazolium chloride. The phage plaques were photographed with a scale, and then the size of the plaques was measured using the scale.

### Measurement of phage infectivity

Plaque-forming ability was determined using a streak test. A dip of phage suspension was streaked onto bacteria-inoculated double-layered agar. After 12 h incubation, plaque forming or “lysis from without” was determined based on the morphology and degree of bacterial inhibition halos formed by the phage. This assay was conducted with all *E. faecalis* strains, and the plaque-forming ability with different *E. faecalis* strains was summarized according to host range.

The plaque-forming ability with various *E. faecalis* strains was quantified by relative EOP. The number of plaques was standardized as one for *E. faecalis* strain EF24, and then the relative EOP of other *E. faecalis* strains was calculated.

The adsorption rate, latent period, and burst size were determined, using previously described methods [26]–[28].

### Phage culture and purification

The phage was cultured with *E. faecalis* strain EF24 in 300 mL of TSB. After the complete lysis of *E. faecalis*, the phage lysate was collected by centrifugation at 10,000×*g* for 10 min at 4°C. Polyethylene glycol 6000 (10%) and 0.5 M NaCl were supplemented to the final concentration and the mixture was left overnight at 4°C. After centrifugation at 10,000×*g* for 40 min at 4°C, the phage pellet was dissolved in TM buffer (10 mM Tris-HCl, pH 7.2; 5 mM MgCl_2_) containing 50 µg mL^−1^ of DNase I (Sigma-Aldrich, St. Louis, MI, USA) and RNase A (Sigma-Aldrich), before incubating at 37°C for 30 min. The phage suspension was then placed on top of a discontinuous cesium chloride (CsCl) density gradient (ρ = 1.3, 1.5, and 1.7) and centrifuged at 50,000×*g* for 2 h at 4°C. The phage bands were collected and purified again using CsCl density gradient ultracentrifugation. The phage band was collected and dialyzed against AAS (0.1 M ammonium acetate, 10 mM NaCl, 1 mM CaCl_2_, 1 mM MgCl_2_, pH 7.2) for 1 h at 4°C.

### Genomic analysis of mutant phage

The genomic DNA of the mutant phage was extracted using a previously described method [17]. Briefly, the purified phage sample containing CsCl was diluted fourfold with AAS and pelleted by ultracentrifugation at 100,000×*g* for 1 h at 4°C. The phage pellet was suspended in protein lysis buffer (1% SDS; 20 mM Tris-HCl, pH 8.0; 50 mM EDTA; 0.2 mg/mL proteinase K, Takara Bio) and incubated for 1 h at 55°C. Phenol extraction and ethanol precipitation were conducted, and then the genomic DNA was solubilized in water.

The whole genome was amplified by PCR with primers (Table S1) using LaboPass SP-Taq (Hokkaido System Science, Hokkaido, Japan), according to the manufacturer's instructions. The PCR products were then purified and sequenced by primer walking with a BigDye terminator v1.1 cycle sequencing kit (Applied Biosystems, Foster City, CA, USA), according to the manufacturer's instructions, using an ABI PRISM 3100-Avant Genetic Analyzer (Applied Biosystems). Sequencing primers are listed in Table S2. Any misreads on the raw data were manually corrected. The sequencing results were assembled using ATGC sequence assembly software (Genetyx Corporation, Tokyo, Japan) and the whole genome was assembled with at least 1× sequence coverage. During mutation site scanning, the mutant phage genome sequence was aligned with the genome sequence of phage ΦEF24C (GenBank accession no. AP009390.1), using ATGC sequence assembly software (Genetyx Corporation). The mutant phage ΦEF24C-P2 genome was deposited into GenBank (accession no. AB609718).

### Bioinformatics analysis of proteins

The theoretical molecular weight (mw) and the isoelectric point (pI) of the proteins were calculated using Genetyx version 10 (Genetyx Corporation). Homology searches in public databases were conducted using the protein Basic Local Alignment Search Tool (BLASTP) at the National Center for Biotechnology Information (NCBI; http://www.ncbi.nlm.nih.gov/). The protein signature was also analyzed with InterProScan (http://www.ebi.ac.uk/Tools/InterProScan/).

The ORF arrangements were compared using Genome Matcher software (http://www.ige.tohoku.ac.jp/joho/gmProject/gmhomeJP.html) [29]. Compared genomes were retrieved from GenBank (*Staphylococcus* phage Twort, AY954970; *Staphylococcus* phage K, AY176327; *Staphylococcus* phage G1, AY954969; *Listeria* phage A511, DQ003638; *Lactobacillus* phage LP65, AY682195; *Bacillus* phage SPO1, FJ230960; *Lactobacillus* phage ΦLb338-1, FJ822135) [30]–[34].

### Cloning and overexpression of the recombinant protein

The coding sequences of *gp31:2640–4150* and *gp31:3680–5200* were amplified by PCR (LaboPass SP-Taq kit) using the appropriate primer sets (Table S3) and with the genomic DNA of phage ΦEF24C as a template, following the manufacturer's instructions. The terminal regions of the PCR products were digested with the restriction enzymes HindIII and SacI (Takara Bio) and cloned into the plasmid pUC18. The accurately cloned fragment was then excised by HindIII and SacI, and recloned into the plasmid pCold II. The plasmids were transformed into *Esc. coli* strains DH5α and BL21 for the purposes of cloning and protein expression, respectively.

Recombinant protein overexpression was conducted using *Esc. coli* BL21 containing an appropriate plasmid, which was grown exponentially to an optical density of 0.5–0.7 at 600 nm, and left for 30 min at 15°C. The growth medium was supplemented with 1 mM isopropyl-β-d-thiogalactopyranoside (IPTG), and bacteria were cultured aerobically for 24 h at 15°C. After centrifugation at 6000×*g* for 10 min at 4°C, the supernatant was decanted and the cell pellet was stored at −80°C until use. To check the overexpression of the recombinant protein in *Esc. coli*, the cell pellet was dissolved in Laemmli's sodium dodecyl sulfate (SDS)-polyacrylamide gel electrophoresis (PAGE) sample buffer, boiled for 5 min, and centrifuged at 20,000×*g* for 5 min at 4°C. The supernatants of IPTG-induced and non-IPTG-induced samples were separately electrophoresed using a 12.5% SDS-PAGE gel and stained using Coomassie brilliant blue R-250 to confirm protein overexpression.

### Purification of the recombinant protein

BL21 (pCold II *gp31:2640–4150*) or BL21 (pCold II *gp31:3680–5200*) were prepared from 250 mL cultures and resuspended in 20 mL lysis buffer (6 M urea, 100 mM sodium phosphate, 300 mM NaCl, pH 7.8) and sonicated (48 pulses×5 s at 5 s intervals) on ice. After centrifugation at 10,000×*g* for 20 min at 20°C, the supernatant was added to 1 mL Talon metal affinity resin (Clontech Laboratories, Mountain View, CA, USA) and gently mixed for 90 min at room temperature. The resin was transferred to a column and was eluted with a wash buffer (6 M urea, 100 mM sodium phosphate, 300 mM NaCl, pH 6.0), followed by the same buffer supplemented with 5 mM imidazole. The elute was collected after the application of 150 mM imidazole-supplemented buffer. Protein purity was analyzed using a 12.5% SDS-PAGE gel with Coomassie brilliant blue R-250 staining.

Protein renaturation was attempted by dialysis. First, the protein was dialyzed against 1 M urea in phosphate buffer saline (PBS, pH 7.2). GP31:2640–4150 was dialyzed against 0.5 M urea in PBS, and GP31:3680–5200 was dialyzed against PBS several times to remove urea. The protein was quantified using a Bradford reagent (Sigma-Aldrich).

### Antiserum of ORF31

Female New Zealand white rabbits (11 weeks, 2 kg) were purchased from SLC Japan (Shizuoka, Japan). The experiments were conducted with the approval of the Animal Experiment Committee of Kochi University (permit no., D-00022). Different rabbits were subcutaneously immunized with 0.5 mg of GP31:2640–4150 in PBS containing 0.5 M urea, or GP31:3680–5200 in PBS, emulsified with complete Freund's adjuvant. Next, the same rabbit was subcutaneously immunized five times at two-week intervals with 0.25 mg of GP31:2640–4150 in PBS containing 0.5 M urea, or GP31:3680–5200 in PBS, emulsified with incomplete Freund's adjuvant. After a sixth immunization with incomplete Freund's adjuvant, the presence of ORF31-specific antibody in the serum was checked by Western blotting against the IPTG-induced BL21 (pCold II *gp31:2640–4150*) or BL21 (pCold II *gp31:3680–5200*). Blood samples were taken one week after the last immunization. The collected blood was incubated overnight at room temperature, and the serum was obtained by centrifugation at 2300×*g* for 15 min at room temperature. The serum was stored at −80°C until use.

### Western blotting

After electrophoresis using a 6% or 12.5% SDS-PAGE gel, the separated proteins were transferred to polyvinylidene fluoride (PVDF) membranes (Immobilon-P Membrane; Millipore, Billerica, MA, USA) using blotting solution (10 mM CHAPS, pH 11; 10% methanol) in a Hoefer TE70 semidry transfer unit (Hoefer Inc., San Francisco, CA, USA) at 1 mA cm^2 −1^ for 90 min. The blotted membrane was blocked with 1% skim milk in PBS, with 0.05% Tween 20 (PBS-T), at 4°C overnight and washed with PBS-T (3×10 min).

The membrane was incubated with either anti-GP31:2640–4150 or anti-GP31:3680–5200 rabbit antiserum, diluted 1∶5000 with 1% skim milk in PBS-T for 1 h at room temperature. The membranes were washed (3×10 min) in PBS-T. The membrane was incubated with a secondary anti-rabbit immunoglobulin G HRP-linked whole antibody (GE Healthcare, Buckinghamshire, UK), which was diluted 1∶100,000 with 1% skim milk in PBS-T, for 30 min at room temperature. The membranes were washed (3×10 min) in PBS-T.

Immunoblot signals were detected using the ECL Plus Western blotting detection system (GE Healthcare) and visualized using X-ray films (Fuji Medical X-ray film, Fujifilm Corporation, Tokyo, Japan).

### Analysis of structural proteins

An equal volume of 2× Laemmli's sample buffer was added to the purified phage in AAS and boiled for 10 min. The phage proteins were electrophoresed using either a 6% or a 12.5% SDS-PAGE gel, with a standard prestained XL-Ladder marker (APRO Life Science Institute, Tokushima, Japan). Structural proteins were visualized by staining the gel with either 2D-silver stain reagent II (Daiichi Pure Chemicals, Tokyo, Japan) or Coomassie brilliant blue R-250. Western blotting was conducted using anti-GP31 rabbit sera.

### Protein concentration

The purified phage in AAS, which was obtained from 1 L culture, was heated for 5 min at 100°C. After cooling to 4°C, the sample was treated with 10 µg mL^−1^ DNase I for 20 min at 37°C. 4× Laemmli's sample buffer was added before heating for 5 min at 100°C. Protein with a molecular weight of more than 100 kDa were concentrated by a centrifugal ultrafiltration using a Vivaspin 4 100000 MWCO (Sartorius Stedim Biotech, Göttingen, Germany) (7000×*g*, room temperature), until the volume was reduced to 400 µL. The supernatant was collected and electrophoresed using a 6% SDS-PAGE gel, before further analysis was conducted.

### N-terminal sequencing

After electrophoresis of the concentrated proteins on a 6% SDS-PAGE gel, the proteins were blotted onto the PVDF membrane (Sequi-Blot PVDF Membrane; Bio-Rad Laboratories, Hercules, CA, USA) using blotting solution (10 mM CHAPS, pH 11; 10% methanol) with a Hoefer TE70 semidry transfer unit (Hoefer Inc.) at 1.2 mA cm^2 −1^ for 180 min at 4°C. The blotted membrane was stained with Coomassie brilliant blue R-250. The targeted protein band was excised from the membrane and sequenced using a PPSQ-31A/33A protein sequencer (Shimadzu, Kyoto, Japan). The amino acid sequence was analyzed by in-house BLASTP search against the phage ΦEF24C genome using In-silico Molecular Cloning genomics edition (In-silico Biology, Yokohama, Japan). The protein sequence was deposited in the UniProt database (accession no. SPIN000005852).

### Matrix assisted laser desorption ionization (MALDI)-time of flight (TOF)/TOF analysis

The concentrated proteins were electrophoresed using a 6% SDS-PAGE gel, and the gel was stained by colloidal Coomassie G-250 staining (0.12% Coomassie brilliant blue G-250, 10% ammonium sulfate, 10% phosphoric acid, 20% methanol) overnight at room temperature [35]. After destaining with MilliQ water, the targeted protein band was excised. The gel was incubated in a destaining solution (200 mM NH_3_HCO_3_, 50% acetonitrile) for 30 min at 37°C, and the destaining solution was replaced. This procedure was repeated until the gel was clear. The gel was then sequentially incubated with a reduction solution (10 mM dithiothreitol, 50 mM NH_3_HCO_3_) for 30 min at 50°C and an alkylating solution (55 mM 2-iodoacetamide, 50 mM NH_3_HCO_3_) for 60 min at 37°C in the dark. After reduction and alkylation of the protein, the gel was rinsed by 50 mM NH_3_HCO_3_ for 10 min at room temperature. The gel was vortexed in 50 mM NH_3_HCO_3_ for 5 min, and then again in acetonitrile for 5 min, before being dried by vacuum centrifugation. The dried gel was soaked in a digestion solution (12.5 ng µL^−1^ trypsin gold, mass spectrometry grade, Promega, Madison, WI, USA; 50 mM NH_3_HCO_3_, 0.1% n-octyl-β-glucoside) on ice for 45 min. The gel was then incubated overnight in 50 mM NH_3_HCO_3_-0.1% n-octyl-β-glucoside at 37°C. The digested peptides were extracted by sonication in 50% acetonitrile and 70% acetonitrile in 0.1% trifluoroacetic acid. The sample was concentrated by vacuum centrifugation and suspended in 5% acetonitrile–0.1% trifluoroacetic acid. The sample was desalted using StageTips (C18 material, 20 µL tip; Proxeon, Odense C, Denmark) and dried by vacuum centrifugation. Samples were dissolved in 5% acetonitrile–0.1% trifluoroacetic acid and cospotted with an equal volume of the matrix (α-cyano-4-hydroxycinnamic acid dissolved in a mixture of 50% acetonitrile–0.1% trifluoroacetic acid in a saturated solution) onto a sample plate.

Finally, the sample was analyzed using an AB SCIEX TOF/TOF 5800 System (AB Sciex, Foster City, CA, USA). The top 20 peaks from the TOF MS analysis were analyzed by tandem mass spectrometry (MS/MS). A protein database for phage ΦEF24C was constructed locally from the phage ΦEF24C genomic data in this study. The MS/MS data were analyzed by Paragon method using the ProteinPilot 3.0 software (AB Sciex) [36].

### Antibody purification

The recombinant protein GP31:3680–5200 was coupled to a Hi-Trap NHS-activated HP column (GE Healthcare) and the antibody purification was conducted according to the manufacturer's instructions. The purity of the antibody was checked by SDS-PAGE, and the protein concentration of the antibody was determined using Bradford reagent. The method obtained 0.4 mg mL^−1^ of purified anti-GP31:3680–5200 antibody.

### Electron microscopy and immunoelectron microscopy

Electron microscopy was conducted by loading a drop of the purified phage ΦEF24C in AAS onto formvar-carbon-coated copper grids for 2 min, and absorbing the phage solution using filter paper (Advantec Toyo, Tokyo, Japan). MilliQ water was then loaded onto the phage-loaded copper grids for 2 min, and the water was again absorbed using filter paper (Advantec Toyo). This treatment with MilliQ water is referred to as a wash in this study, and two washes were conducted after phage loading. The copper grids were then negatively stained with 2% uranyl acetate (pH 4.0).

Immunoelectron microscopy was conducted by incubating the purified phage with anti-GP31:3680–5200 antibody diluted with PBS (1∶100) for 30 min at room temperature. The samples were then loaded onto formvar-carbon-coated copper grids. Each copper grid was washed once with MilliQ water, and then incubated with 12 nm colloidal gold-AffiniPure goat anti-rabbit IgG (H+L) (Jackson ImmunoResearch Laboratories, West Grove, PA, USA) in PBS (1∶50) for 30 min at 37°C. Each grid was then washed twice with MilliQ water and negatively stained with 2% uranyl acetate (pH 4.0).

Electron micrographs were acquired using a Hitachi H-7100 transmission electron microscope (Hitachi, Ibaraki, Japan) at 100 kV.

### Measurements of therapeutic efficacy in vitro

*E. faecalis* strain EF11 at mid-log phase was washed with saline three times. The bacteria were suspended in saline, and bacterial concentration (bacteria mL^−1^) was determined by turbidity (in Klett units), measured using a Klett-Summerson photoelectric colorimeter (Klett Manufacturing Co., NY, USA). Phage was prepared at an appropriate concentration, diluted with TSB.

A bacterial suspension was prepared at a concentration of ca. 3.0×10^9^ bacteria mL^−1^. The phage suspension was ca. 3.0×10^7^ pfu mL^−1^. Five microliters of bacterial suspension and 5 µL of phage suspension were mixed with 140 µL TSB in one well of a 96-well microtiter plate. The plate was incubated with shaking at 37°C and the turbidity was measured every 15 min at 600 nm, using a HiTS incubation reader (Scinics Corporation, Tokyo, Japan).

A bacterial suspension of ca. 1.5×10^10^ bacteria mL^−1^ was prepared. Phage was diluted with TSB and a suspension of ca. 1.5×10^8^ pfu mL^−1^ was prepared. Human blood from a healthy male volunteer donor was collected into a heparinized blood collection tube. Five microliters of the bacterial suspension and 5 µL of phage suspension were mixed with 900 µL of human blood, and the mixture was incubated. After incubation for one day at 37°C, the blood culture was diluted and plated. After incubation overnight, a colony count was conducted.

### Statistical analysis

All statistical analyses were performed using GraphPad InStat version 3 (GraphPad Software Inc., La Jolla, CA, USA).

## Results

### Large-plaque-forming spontaneous mutant phage ΦEF24C-P2

**Figure 1:**
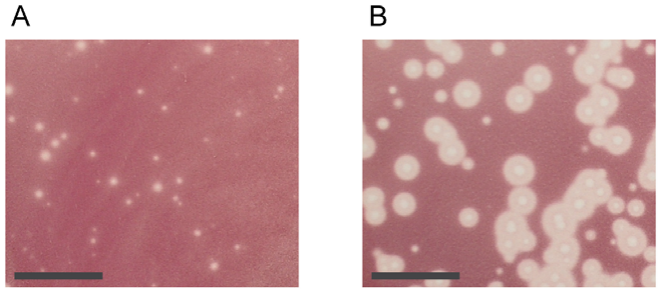
Plaque formation of wild-type phage ΦEF24C and mutant phage ΦEF24C-P2 with *E. faecalis* strain VRE2. (A) Plaques formed by wild-type phage ΦEF24C. (B) Plaques formed by mutant phage ΦEF24C-P2. The bar indicates 10 mm. Photographs were taken after incubation for 12 h at 37°C. Mutant phage ΦEF24C-P2 formed larger plaques than wild-type phage ΦEF24C.

Phage ΦEF24C was plated onto *E. faecalis* strain VRE2 and a large-plaque-forming spontaneous mutant phage ΦEF24C-P2 was isolated (Figure 1). The plaque sizes of wild-type phage ΦEF24C and mutant phage ΦEF24C-P2 were 0.74±0.33 mm and 3±1.21 mm (mean ± SD; n = 20), respectively. The plaque sizes formed by phages ΦEF24C and ΦEF24C-P2 were significantly different (Mann-Whitney U test; *P*<0.0001). We hypothesized that the difference in the plaque size was because of the improved infectious ability of mutant phage ΦEF24C-P2.

Host range and phage infectivity were compared with various clinical *E. faecalis* strain isolates to determine whether the mutant phage had enhanced infectious ability. There was no difference in the host range between the wild-type phage ΦEF24C and the mutant phage ΦEF24C-P2. On the other hand, phage infectivity with various bacterial strains was evaluated by comparing the EOP of the wild-type phage ΦEF24C and the mutant phage ΦEF24C-P2. *E. faecalis* strain EF24 was used as a reference strain in the EOP assay, because phages ΦEF24C and ΦEF24C-P2 were adapted to this bacterial strain and formed clear countable plaques with this strain. The mutant phage ΦEF24C-P2 had an improved EOP compared with the wild-type phage ΦEF24C (Table 1). The mutant phage ΦEF24C-P2 showed a tenfold increase in EOP values when compared with bacterial strains that were mainly isolated from urine in this study.

### Improved phage adsorption

Enhancement of key stages affecting the phage life cycle, i.e., adsorption, latent period, and burst size, were likely to account for the greater infectivity of phage ΦEF24C-P2 [37]. Previous studies using *E. faecalis* strain EF24 as a reference strain found that the adsorption rate was 90% in 5 min. The latent period and the burst size of phage ΦEF24C are known to be ca. 30 min and ca. 110–120, respectively. The latent period and burst size can be evaluated by comparing the phage production rate 55 min post infection between wild-type phage ΦEF24C and mutant phage ΦEF24C-P2. Therefore, the phage adsorption rate at 5 min and the phage production rate 55 min post infection were compared for the wild-type phage ΦEF24C and the mutant phage ΦEF24C-P2, using *E. faecalis* strain EF24.

**Figure 2:**
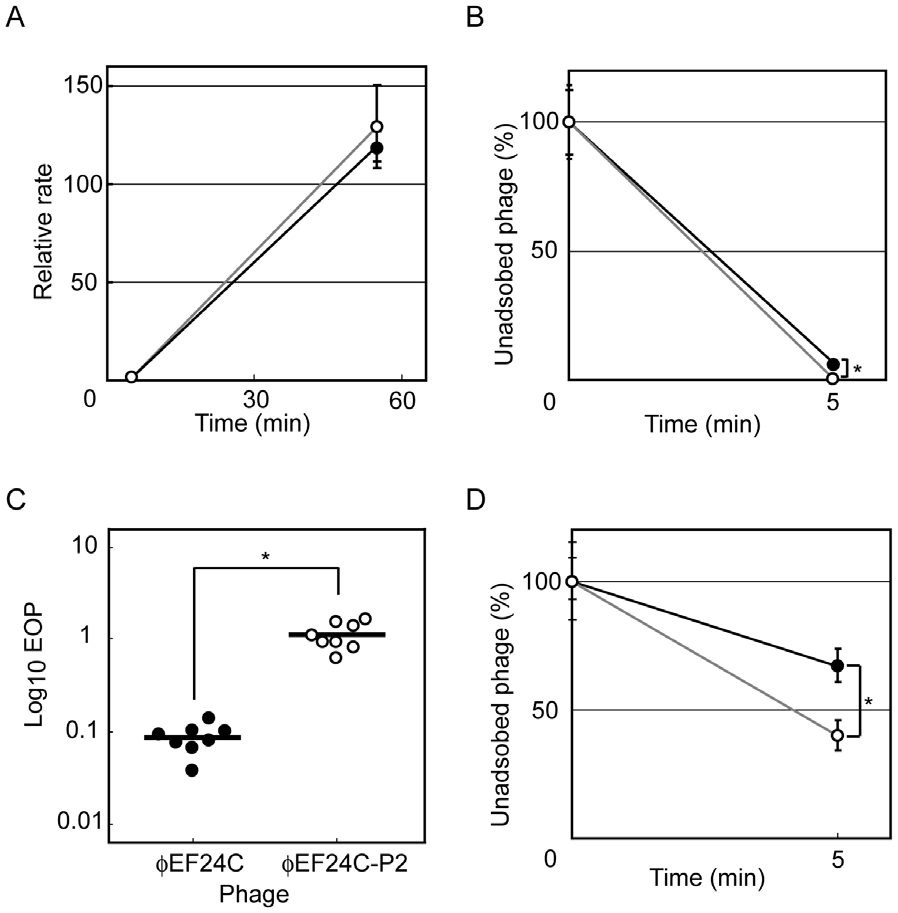
Comparison of the infectivity of phage ΦEF24C and phage ΦEF24C-P2. • and ○ indicate wild-type phage ΦEF24C and mutant phage ΦEF24C-P2, respectively. On the graphs, error bars indicate SD. Statistically significant differences are indicated by an asterisk. (A) Phage production 55 min post infection of *E. faecalis* strain EF24. Phages ΦEF24C (n = 6) and ΦEF24C-P2 (n = 6) were not significantly different (Mann-Whitney U test; *P* = 0.3939). (B) Phage adsorption in 5 min to *E. faecalis* strain EF24. Phages ΦEF24C (n = 6) and ΦEF24C-P2 (n = 6) were significantly different (Mann-Whitney U test; *P*<0.005). (C) EOP with *E. faecalis* strain EF11. Phages ΦEF24C (n = 8) and ΦEF24C-P2 (n = 8) were significantly different (Mann-Whitney U test; *P*<0.0005. (D) Phage adsorption in 5 min to *E. faecalis* strain EF11. Phages ΦEF24C (n = 6) and ΦEF24C-P2 (n = 6) were significantly different (Mann-Whitney U test; *P*<0.005).

The phage production rates 55 min post infection did not differ significantly between the wild-type phage ΦEF24C and the mutant phage ΦEF24C-P2 (Figure 2A). By contrast, adsorption rates 5 min after phage inoculation were significantly different between the wild-type ΦEF24C and the mutant phage ΦEF24C-P2 (Figure 2B), although the difference was small.

The improved adsorption efficiency was confirmed by testing phage ΦEF24C-P2 adsorption in 5 min using *E. faecalis* strain EF11, which is a bacterial strain with a lower EOP, i.e., more sensitive to phage ΦEF24C-P2 than to phage ΦEF24C (Figure 2C and 2D). The adsorption efficiency of phage ΦEF24C-P2 was significantly higher than that of phage ΦEF24C. Thus, the mutant phage ΦEF24C-P2 was considered to have an improved adsorption efficiency.

### A spontaneous point mutation found on a large putative gene

**Figure 3:**
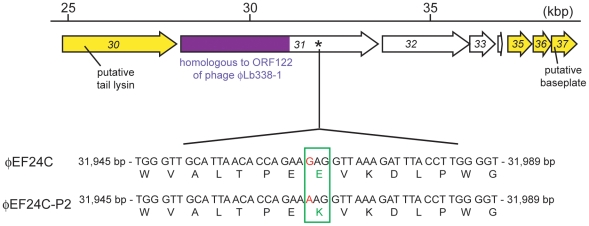
Mutation site in *orf31* of phage ΦEF24C-P2 and the features of ORF31 and neighboring ORFs. The mutation site is shown in the DNA alignments between phage ΦEF24C and phage ΦEF24C-P2, in the bottom row. A point mutation (i.e., substitution) was found on the putative gene *orf31*, where the nucleotides are shown in red. The point mutation changed an amino acid from glutamic acid (negatively charged amino acid; wild-type phage ΦEF24C) to lysine (positively charged amino acid; mutant phage ΦEF24C-P2), which is colored and boxed in green. The partial genomic map of phage ΦEF24C is shown in the top row. The arrows indicate each ORF. The arrows in yellow indicate ORFs with similarity to other phage proteins. The region colored in purple, the N-terminal region of the ORF31, is similar to the N-terminal region of ORF122 of *Lactobacillus paracasei* phage ΦLb338-1, with an E-value of 1.0×10^−114^ in the BLASTP analysis. It occupied 53% of the ORF31 protein sequence. A mutation site is denoted by an asterisk around the C-terminal region of the ORF31, but no protein signature was found. ORF32–34 did not show similarity to the other proteins.

The whole genome sequence of the mutant phage ΦEF24C-P2 was screened to search for the causative genetic factor. Only a single point mutation in the nucleotide 31,966 on *orf31* was discovered (Figure 3) by comparing the whole genome sequence for the mutant phage ΦEF24C-P2 and the wild-type phage ΦEF24C. The guanine (G) residue at nucleotide 31,966 in the wild-type phage ΦEF24C was changed to adenine (A) in the mutant phage ΦEF24C-P2, which would result in replacing a single negatively charged glutamic acid (E) in ORF31 of the wild-type phage ΦEF24C with a positively charged lysine (K) in the mutant phage ΦEF24C-P2 (Figure 3).

### Bioinformatics analysis of the putative ORF31 protein

ORF31 was predicted to be a large protein composed of 1825 amino acids (theoretical mw, 203 kDa; theoretical pI, 4.7). There had been frequent data updates in GenBank since the first report of the phage ΦEF24C genome, so the BLASTP analysis of ORF31 was conducted again. Overall, the N-terminal region of ORF31 was similar to the N-terminal region of hypothetical proteins with unknown function in most SPO1-like viruses. Among them, only *Lactobacillus* phages ΦLb338-1 and LP65 seemed to have similar large proteins: ORF122 (theoretical mw, 235 kDa; theoretical pI, 4.82) and ORF97 (theoretical mw, 221 kDa; theoretical pI, 4.91), respectively [30], [31]. 52% of the ORF31 at the N-terminal showed similarity to ORF122 of *Lactobacillus* phage ΦLb338-1, with an E-value of 2e-114, whereas 16% of the ORF31 at the N-terminal showed similarity to ORF97 of *Lactobacillus* phage LP65, with an E-value of 8e-13 (Figure 3 and Figure S1). The protein sequence, mw, and pI of the protein were similar, so ORF31 may be functionally similar to ORF122 of *Lactobacillus* phage ΦLb338-1.

No significant protein domains was not predicted by InterProScan and no similar proteins with known function were found by the BLASTP search of the NCBI, so the function of ORF31 was difficult to predict. Thus, the similar ORF122 protein found in phage ΦLb338-1 was analyzed to facilitate protein function prediction (Figure S1). The C-terminal region of phage ΦLb338-1 ORF122 was predicted by bioinformatics to have not only the galactose binding domain-like superfamily and similar protein sequences to the putative host specificity proteins. Taking into account the mw, bioinformatics analysis, and the association of ORF31 with adsorption in the mutant phage analysis, it was hypothesized that ORF31 could be a component of a tail fiber.

### Identification of ORF31 as a phage structural protein

**Figure 4:**
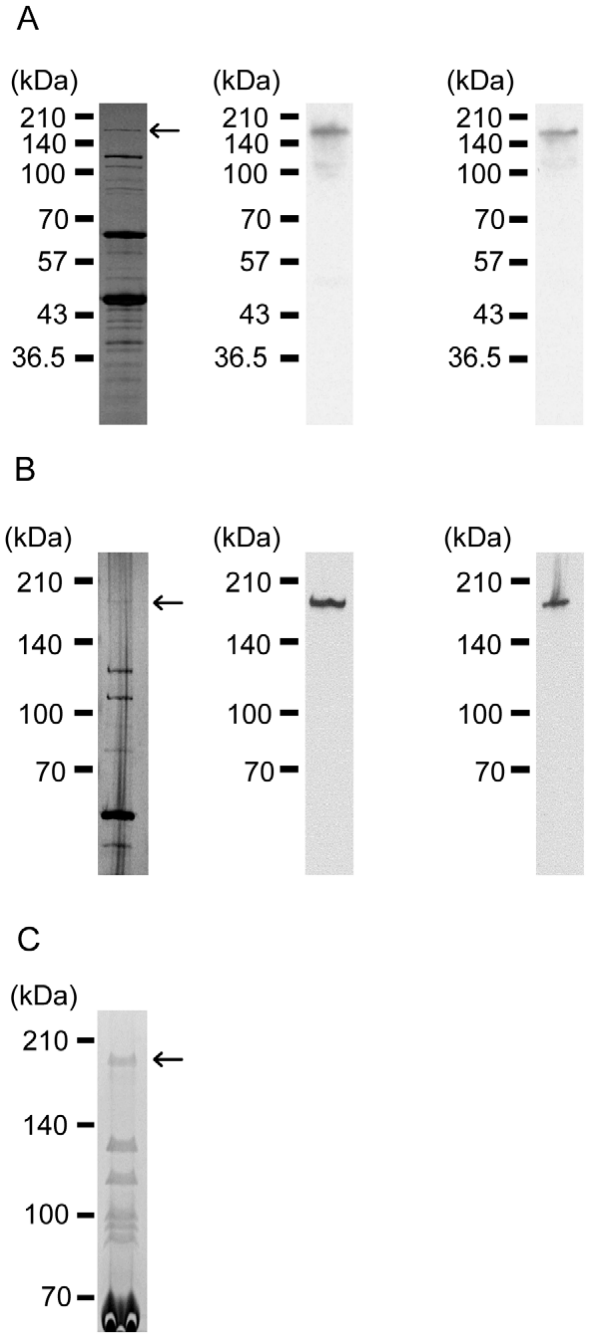
Structural protein analysis. Structural protein analysis and Western blot with anti-ORF31 rabbit antisera, separated using (A) 12.5% and (B) 6% SDS-PAGE gels. SDS-PAGE images of the structural proteins visualized by silver staining are shown in the left column. The middle and right columns show the Western blot images created by anti-GP31:2640–4150 and anti-GP31:3680–5200 rabbit antisera. The arrows indicate the possible ORF31 protein band detected by the Western blot analysis. (C) SDS-PAGE image of the concentrated phage ΦEF24C proteins. Phage ΦEF24C proteins were concentrated by 100 kDa cutoff centrifugal ultrafiltration. The phage proteins were electrophoresed using a 6% SDS-PAGE gel and visualized by colloidal Coomassie G-250 staining. The arrow indicates the protein analyzed by MALDI-TOF/TOF and N-terminal sequencing (see Figures S3 and S4).

ORF31 was annotated as a large protein, and the bioinformatic analysis suggested that it could be a tail fiber. However, it was not certain if such a large protein was present in the structural proteins. Moreover, if present, mRNA splicing or posttranslational modification could occur and the mw of the protein might be significantly smaller than predicted. Thus, Western blotting was conducted to test for the presence of ORF31 in the phage structural proteins with the predicted mw. Before the start of the experiment, anti-ORF31 sera were produced by immunization of rabbits with the recombinant proteins GP31:2640–4150 and GP31:3680–5200 (Figure S2). The anti-GP31:2640–4150 or anti-GP31:3680–5200 rabbit serum was used in Western blotting, which was performed with phage ΦEF24C structural proteins separated on 12.5% and 6% SDS-PAGE gels. The results of the Western blot analysis showed that the ORF31 was present as a protein of ca. 183 kDa. Comparison of the mw detected in the Western blot with the mw of the structural proteins in the SDS-PAGE analysis showed that the protein detected in the Western blot corresponded to the top protein band detected in only the SDS-PAGE gel stained by silver staining (Figure 4A and 4B). However, the protein was barely detected by Coomassie brilliant blue R-250 staining. Thus, the protein was considered to be expressed in very small quantities relative to other ΦEF24C structural proteins.

To validate the presence of ORF31 in the structural proteins, the protein detected by the Western blot was analyzed further. The low levels of the target protein meant that the structural proteins had to be concentrated by centrifugal ultrafiltration before analysis by MALDI-TOF/TOF and N-terminal sequencing (Figure 4C). After separation of the concentrated structural proteins using a 6% SDS-PAGE gel, the target protein was digested with trypsin and analyzed by tandem TOF MS. As a result, the protein was strongly suggested to be ORF31 (Figure S3). A difference of approximately 20 kDa was seen between theoretical calculations (ca. 203 kDa) and experimental estimates of the mw (ca. 183 kDa). Phage proteins are frequently N-terminally processed and a start codon of the *orf* could be mistakenly annotated, so the protein was N-terminally sequenced. In-house BLASTP analysis of the N-terminal protein sequence of ORF31 showed that the protein sequence matched the sequence of the putative ORF31 from amino acids 5–14. Thus, ORF31 was considered to be encoded by *orf31* as formally predicted (Figure S4). The first four amino acids at the N-terminal end appeared to be processed or degraded. Thus, ORF31 was considered to be encoded exactly as predicted using bioinformatics, and the difference between the theoretical calculation and the experimental calculation of the mw was probably attributable to the behavior of such a large protein on the SDS-PAGE.

### Tail fiber of phage ΦEF24C

**Figure 5:**
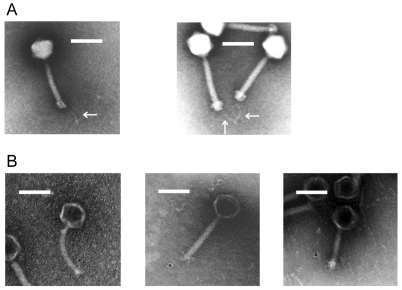
Electron microscopic analysis of phage ΦEF24C. (A) Electron microscopic analysis of phage ΦEF24C tail fiber. A single thin tail fiber with a knob was observed at the tail end of phage ΦEF24C. The length of the tail fiber was found to be 78.6±4.2 nm (n = 8; mean ± SD). In the left electron micrograph, a tail fiber was flexibly extended from the tail of phage ΦEF24C. In the right electron micrograph, the tips of the tail fibers appear to bind each other. Bars indicate 100 nm. Arrows indicate tail fibers. (B) Immunoelectron microscopic analysis of the ORF31 of phage ΦEF24C. Phage ΦEF24C was initially treated using the anti-GP31:3680–5200 antibody, and then treated with the immunogold-conjugated antibody. Gold particles were detected around the tail end. In the left micrograph, a gold particle is located on the tail fiber of phage ΦEF24C. In the middle and right micrographs, a gold particle seems to be located around the tail, but the tail fiber is not clearly visible. Bars indicate 100 nm.

The large structural protein ORF31 was found to be associated with adsorption, and bioinformatics analysis indicated that it was a tail fiber. Thus, it was hypothesized that a tail fiber might be present in phage ΦEF24C. However, a tail fiber was not observed using conventional methods [16]. Thus, attempts were made to observe the phage ΦEF24C tail end. Following the additional wash step after loading the sample onto the formvar-coated copper mesh, a flexible long tail fiber extending from the tail end was observed in phage ΦEF24C (Figure 5A).

### ORF31 as a possible component of a tail fiber

Using the purified anti-GP31:3680–5200 antibody, immunoelectron microscopy was conducted. Several sample treatments were involved in the immunoelectron microscopy, and the immunogold-attached fine tail fiber of phage ΦEF24C was difficult to observe. However, some electron micrographs showed a tail fiber attached to a gold particle (Figure 5B). Therefore, these facts strongly suggested that ORF31 was a component of the tail fiber.

### Improvement of in vitro therapeutic efficacy

**Figure 6:**
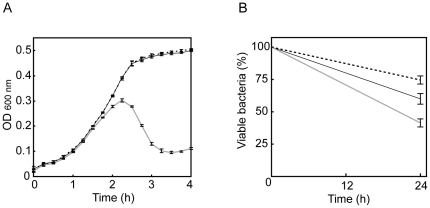
The improved infectivity of phage ΦEF24C-P2. (A) Lytic activities of phages ΦEF24C and ΦEF24C-P2 in TSB media. *E. faecalis* strain EF11 was incubated with/without phage at MOI 0.01. Changes in bacterial concentration were tracked over time and measured by optical density at 600 nm. The mean value is plotted, and the SD is shown as an error bar (n = 3). (B) Antimicrobial activities of phages ΦEF24C and ΦEF24C-P2 in human blood. *E. faecalis* strain EF11 was incubated with/without phage at MOI 0.01 for 24 h at 37°C, in human blood. After incubation, viable bacteria were measured by colony counting. The bacterial concentration changes are shown as percentages. Error bars indicate SD (n = 6). Two groups of data sets were analyzed with the Mann-Whitney U test. The bacterial concentration was significantly reduced by the wild-type phage ΦEF24C (*P*<0.05) and the mutant phage ΦEF24C-P2 (*P*<0.005) compared with that in the control group. Moreover, compared with the phage-ΦEF24C-treated group, the phage-ΦEF24C-P2-treated group showed a significant reduction in the bacterial concentration (*P*<0.01). The gray line indicates bacterial growth in the presence of phage ΦEF24C-P2. The black line indicates bacterial growth in the presence of phage ΦEF24C. The dotted line indicates bacterial growth without the phage.

Phage ΦEF24C has previously demonstrated in vivo therapeutic effects, so phage ΦEF24C mutants with sufficient infectivity are expected to be therapeutically effective in vivo. Thus, the bactericidal effects of phages ΦEF24C and ΦEF24C-P2 against *E. faecalis* strain EF11, which was less sensitive to phage ΦEF24C, were measured in vitro. Phages ΦEF24C and ΦEF24C-P2 were cultured with *E. faecalis* to yield a multiplicity of infection (MOI) of 0.01 in TSB media. Phage ΦEF24C-P2 lysed bacteria within 3 h, whereas phage ΦEF24C did not (Figure 6A). Next, phage was cultured with bacteria to yield an MOI of 0.01 in human blood. Phages ΦEF24C and ΦEF24C-P2 both successfully reduced bacterial concentration in human blood after incubation for 24 h compared with no phage treatment, but the mutant phage ΦEF24C-P2 showed a significantly greater reduction in the bacterial concentration compared with the wild-type phage ΦEF24C after incubation for 24 h (Figure 6B). This result suggests that the bactericidal activities of both phages were not affected by the biological factors in the blood. Thus, the mutant phage ΦEF24C-P2 was considered to have improved therapeutic effects over the wild-type phage ΦEF24C, even in human blood.

## Discussion

Phage ΦEF24C-P2 was investigated because of the great current interest in therapeutic phage development. Whole genome sequence scanning detected a point mutation in *orf31*. This study subsequently elucidated the presence of a tail fiber in phage ΦEF24C and showed that ORF31 was a component of the long tail fiber. SPO1-like viruses are important as therapeutic phages. Tail fiber genes can be useful targets for genetic recombination in future therapeutic phage development, so the tail fiber genes of SPO1-like viruses are of interest. However, the putative proteins of other SPO1-like viruses corresponding to ORF31 only showed similarity to the N-terminal region of the ORF31, and their molecular sizes were much smaller than that of ORF31 in some cases. In addition, it was not known whether this long tail fiber was unique to phage ΦEF24C. A review of previous studies showed that two types of tail fibers were shown by electron microscopic studies of the SPO1-like viruses: short tail fibers around the tail edge, and a single long tail fiber extending from the tail end [14], [31], [32]. Several short tail fibers have been seen in the *Listeria* phage A511, but a long tail fiber has been reported only in the *Lactobacillus* phage LP65 [31], [32].

**Figure 7:**
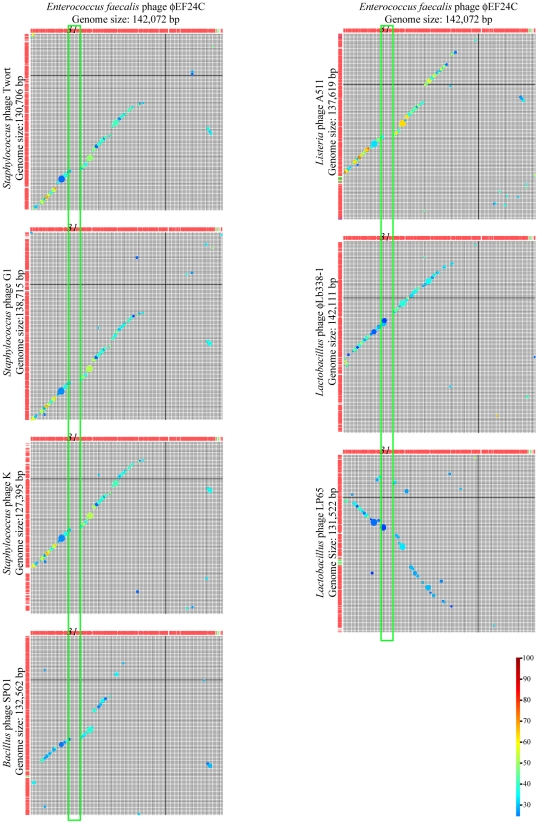
Reciprocal genomic comparison of phage ΦEF24C with other SPO1-like viruses. Whole genome matrix analysis by BLASTP assessed the arrangement of the similar ORFs, using Genome Matcher (http://www.ige.tohoku.ac.jp/joho/gmProject/gmhomeJP.html) [29]. The phage ΦEF24C genome and other compared genomes are shown in the rows and the columns of each figure, respectively. The genomic direction is shown left to right and top to bottom. Similarity is shown by size and the color of the circle. The degree of similarity is shown in color and a colored scale bar is shown on the bottom right. In the top row of matrices, the ORF31 of phage ΦEF24C is numbered *31*. The green boxes indicate the structural protein region, including ORF31, but without similarities. Fifty-seven percent of the phage ΦEF24C genome was conserved in the ORF arrangement compared with the SPO1-like viruses. In ORF31, a different level of similarity was observed. However, ORF122 of *Lactobacillus* phage ΦLb338-1 and ORF97 of *Lactobacillus* phage LP65 were similar to ORF31 in terms of molecular size and N-terminal protein sequence. No similarity to other ORFs of the SPO1-like viruses was observed for ORF32–ORF34.

The essential proteins such as major structural proteins are fairly conserved in the related phages, and functionally related genes are closely aligned [38]–[40]. However, adsorption proteins such as tail fiber proteins are expected to be diverse because they respond to the various surface structures of bacterial species [41]–[44]. Thus, a whole genome comparison of SPO1-like viruses was conducted using BLASTP to estimate the presence of the genes encoding for a tail fiber. According to the reciprocal genomic comparison by BLASTP between SPO1-like viruses (Figure 7), the homologous ORFs were clustered and the ORF arrangements were also conserved. These conserved ORFs occupied 57% of the ORFs on the phage ΦEF24C genome and were considered to be essential in the phage structure. When the homologous proteins were continuously aligned between the phage genomes, the ORF arrangements were linear, as can be seen in the matrices shown in Figure 7. However, a nonhomologous region was present between ORF31 and ORF34, which is shown as a gap in the matrices in Figure 7. This gap in the linearity of the ORF arrangement around ORF31 to ORF34 suggests that the genes corresponding to *orf31* to *orf34* appeared to be present in *Lactobacillus* phages LP65 and ΦLb338-1, but missing in other SPO1-like viruses. This suggests that the long tail fiber is likely to be present in *Enterococcus* phages ΦEF24C and *Lactobacillus* phages LP65 and ΦLb338-1. Therefore, it was concluded that some SPO1-like viruses might have a long tail fiber.

In T-even phages such as phages T2 and T4, the tip of the long tail fibers (i.e., the ligand) binds to the first molecules on the bacterial surface (i.e., a receptor), and the tail pin is then irreversibly attached to a second molecule on the bacterial surface (i.e., a receptor). Phages T2 and T4 have tail fibers that are similar in shape, but the ligand proteins of these tail fibers differ in molecular weight and their function is slightly different. In phage T4, one of the largest proteins, gp37, functions as both a tail fiber shaft and a ligand protein [45], [46]. In phage T2, a small protein, gp38, binds to a tip of the long tail fiber and only functions as a ligand protein [22], [25]. In phage ΦEF24C, the ligand protein genes are still unknown. This study and the reciprocal genome study suggest that ORF31 and related proteins might be a ligand component of tail fibers. Thus, recombinant proteins of ORF31 to ORF34 might affect their binding ability. Recombinant proteins of intact ORF31 and intact ORF32 were produced with some effort, but these recombinant proteins were not expressed in *Esc. coli* (data not shown). Subsequently, recombinant proteins of partially deleted ORF31, partially deleted ORF32, and intact ORF33 were produced. However, these were insoluble, or they were soluble but did not show binding activity to *E. faecalis* (data not shown). Thus, further study is required to identify the ligand part of the tail fiber. A ligand protein might require a chaperone protein to fold correctly as seen in phage T4 [45], [46], or a heteromultimer may function as the ligand.

Such an increase in phage infectivity caused by a mutation is not an unusual event. Phage and bacteria evolve in the context of a coevolutionary arms race between the host cell defenses and the phage counterdefenses, and the population dynamics of the phage and the host bacteria can propel molecular evolution toward adaptation and counteradaptation [47], [48]. During the short-term coevolution of bacteria and phages, bacteria modify their surface structures, and phages tend to change their adsorption molecules [49], [50]. In this study, a phenotypically altered phage was produced that managed to infect a bacterial strain that was normally less sensitive to phage, during the short-term coevolution of the phage and the bacterium. Therefore, such a coevolutionary study of the relationship between the host cell defenses and the phage counterdefenses should benefit the development of a therapeutic phage and facilitate the future introduction of phage therapies to Western countries.

Finally, the benefits of phage ΦEF24C-P2 in therapeutic use were examined. The in vivo life-saving effects of phage ΦEF24C were previously demonstrated, so in vivo therapeutic effects were also expected of phage ΦEF24C-P2. In this study, phage ΦEF24C-P2 showed significantly higher antimicrobial effects in TSB media and human blood compared with phage ΦEF24C, particularly against bacterial strains derived from urological infections (the type of infection was unknown). The bacteriolytic activity of the wild-type phage ΦEF24C seemed to be higher in human blood than in TSB medium (Figure 6). Although the mechanisms underlying this observation are not known, some unknown factors may increase the infectivity, especially the adsorption ability, of the phage in human blood. Urological infections caused by *Enterococci* are generally difficult to treat, because the biofilm formed by enterococcal cells impedes drug access to the site of infection [51]. Thus, there are significant challenges for the treatment of enterococcal infections in clinical settings. Fortunately, phage therapy has recently proved its effectiveness against enterococcal chronic prostatitis in clinical case studies [52], [53]. Thus, phage ΦEF24C-P2 might be a useful therapeutic phage against such urological infections.

Tail fibers could be good targets for phage genetic modification in therapeutic phage development, so the characterization of tail fibers is considered important. The ligand part of the tail fiber has not been identified, but this study suggested that ORF31 may be a good target for phage genetic modification. Despite the long history of research and its importance in phage therapy, this is the first report of molecular characterization of a long tail fiber in SPO1-like viruses.

## Supporting Information

Figure S1 **Bioinformatics analysis of ORF31 of phage ÖEF24C and ORF122 of** ***Lactobacillus paracasei*** **phage ΦLb338-1.** At the N-terminal end, 53% of the ORF31 sequence was similar to *Lactobacillus* phage ΦLb338-1 ORF122, with an E-value of 1.0×10^−114^. No protein signature was found for ORF31. The mutation site found in phage ΦEF24C-P2 is shown by an asterisk. By contrast, the BLASTp on NCBI showed that the ORF122 of *Lactobacillus paracasei* phage ΦLb338-1 was similar to the putative tail host specificity proteins from phages or prophages of intestinal bacteria in the C-terminal region (approximately from amino acid 2000 to 2222). InterProScan predicted a galactose binding domain-like superfamily for amino acids 1463–1606 in the *Lactobacillus* phage ΦLb338-1 ORF122.
(EPS)

Figure S2 **The recombinant proteins of ORF31.** Two recombinant proteins, GP31:2640–4150 and GP31:3680–5200, were produced in *Esc. coli*. The mutation site found in phage ΦEF24C-P2 is denoted by an asterisk. The form of the protein (soluble or insoluble) is shown on the right.
(EPS)

Figure S3 **MALDI-TOF/TOF analysis of the ORF31 protein identified by western blotting.** The concentrated protein, indicated by an arrow in Figure 5C, was analyzed by MALDI-TOF/TOF. (A) Mass spectrum of the trypsin-digested protein. Peptide peaks were are numbered on the top, were further analyzed. (B) Summary of the MS/MS data. The data acquired from each peak were analyzed by Paragon method using ProteinPilot 3.0 software (AB Sciex) against the phage ΦEF24C database. The protein was strongly suggested to be ORF31 (Unused ProtScore, 28; total ProtScore, 28; sequence coverage, 16.6%; No. of pepetide with 95% confidence, 14).
(EPS)

Figure S4 **N-terminal sequence of ORF31.** The concentrated protein indicated in Figure 5C was N-terminally sequenced. The putative ORF31 and N-terminal sequences of the ORF31 are shown at the top and bottom, respectively. Amino acids are shown as a single letter. X indicates an unknown. The N-terminal amino acid sequence was analyzed against the putative ORF31 by in-house BLASTp search using Genetyx version 10 (Genetyx Corporation). The matched sequence is highlighted in gray. ORF31 appeared to start from the amino acid 5 of the putative ORF31. The first four amino acids from the N-terminal may be posttranslationally processed, or might be lost by degradation of the protein.
(EPS)

Table S1 **PCR primers for identifying genomic fragments.**
(PDF)

Table S2 **List of genome sequencing primers.**
(PDF)

Table S3 **Primers used for cloning the genes of partial** ***orf31*** **.**
(DOC)
