# Supplementary material for: Improved Adsorption of an Enterococcus faecalis Bacteriophage ΦEF24C with a Spontaneous Point Mutation
Source: PLoS One. 2011 Oct 25;6(10):e26648. doi: 10.1371/journal.pone.0026648 (PMC3201976; doi:10.1371/journal.pone.0026648)
Supplement: Table S1 — PCR primers for identifying genomic fragments. (PDF) [file pone.0026648.s005.pdf]

Table S1. PCR primers for amplifying genomic fragments.

Primer	Sequence	Position		Direction[a]	PCR product
		Start	end		
F1K	GGAACTCTACCTGCACTTACATCTGAGC	917	944	F	F1K + R8K PCR
R8K	CTATGTCTGCTCTAGGGTTCCTAACAGC	8333	8306	R	F1K + R8K PCR
F8K	GCCCGCAAGAAACTCTGAAACAGGTG	7924	7949	F	F8K + R15K PCR
R15K	CGTGTACCTTTACCCCAACTCTGTGG	15304	15279	R	F8K + R15K PCR
F15K	GGGTCTTCTGTACAGAGAATGCCTAGC	14958	14984	F	F15K + R21K PCR
R21K	CTCTGTTTAGTTCCTGTTGTGCCCTAGAG	21618	21590	R	F15K + R21K PCR
F20K	GTAGAGGTGCCAGAAGTACTAGCTGATG	20361	20388	F	F20K + R26K PCR
R26K	CTGTTTAGCTGTTGAGCCATCGCTCTC	25957	25931	R	F20K + R26K PCR
F25K	CAATAACGGTGAAGCAGAGAAGGTAAGC	24946	24973	F	F25K + R31K PCR
R31K	GACCCTGTAAATCGTTAGCCCAAGCAGTG	31693	31665	R	F25K + R31K PCR
F30K	GAGCGACATTCTCGGACTATTACGCAAG	29975	30002	F	F30K + R36K PCR
R36K	GGAGAAGGTTAAAACCCACCCGTTTCG	35892	35866	R	F30K + R36K PCR
F35K	GGAGCACCTGCTGTAGAATTAGGAGC	34981	35006	F	F35K + R41K PCR
R41K	CCTCTGTGCCTTCCTCTAACTTGGGAAC	41094	41067	R	F35K + R41K PCR
F40K	GGACTACAGACCTGCAGGCATTAAACTG	39821	39848	F	F40K + gp41R PCR
gp41R	GTCTTCTTACTCTTGGACGGAGGAA	45662	45638	R	F40K + gp41R PCR
Sg2to3p11	CGTGGAATATCAGAAGGTGGGC	44711	44732	F	Sg2to3p11 + R46K
R46K	CGCAGGGCTGAATTGGTATCCATCTG	46360	46335	R	Sg2to3p11 + R46K
F46K	CTAAGGTGGGTATGTCAAGGAGCATG	46165	46190	F	F46K + R53K PCR
R53K	GCTCTTGCTTGCTCTAACAGAGGAG	53435	53411	R	F46K + R53K PCR
F53K	GCTACCTATGTGGTGCTTTATTGAGCC	53288	53314	F	F53K + R60K PCR
R60K	GAACAGTTTTCACATCACCTGAACTACTG	60153	60125	R	F53K + R60K PCR
F60K	CAGAGATGCTCAGCTAATAGAAATACCAG	60002	60030	F	F60K + R68K PCR
R68K	GGTTGTAGTGAACTAGGTGTACCACTG	68033	68007	R	F60K + R68K PCR
F68K	GCGCAAAGGACTAGCACCTAACCC	67923	67946	F	F68K + R76K PCR
R76K	CGTTATACCCACAACAGTCTGGCTCTG	75861	75835	R	F68K + R76K PCR
F1RVr5	CAACGTGAGGGAGCTTATCTTG	72505	72526	F	F1RVr5 + F1RVr9p3R5 PCR
F1RVr9p3R5	GAAACTTTGGGAGCTTGTGGC	77395	77375	R	F1RVr5 + F1RVr9p3R5 PCR
F1RVr9	GTTAGGTGAGTATAAACAACCTAGCCGTT	74826	74854	F	F1RVr9 + F1RVr9p3R PCR
F1RVr9p3R	CTCCTCTTAGGTCTTCAATAGCATC	81526	81502	R	F1RVr9 + F1RVr9p3R PCR
F1RVr9p3	GCGTAAGACACAAGGCGCAGAACCT	81324	81348	F	F1RVr9p3 + F1RVr9p4SR1 PCR
F1RVr9p4SR1	GCCACTACAACTGTTAATGTTACAC	87362	87338	R	F1RVr9p3 + F1RVr9p4SR1 PCR
F87K	GTAGCAGGCTACACAGAGTCTACTTC	87199	87224	F	F87K + R95K PCR
R95K	CCCTCGTAACCACCCATTCAGTTCTTC	94711	94685	R	F87K + R95K PCR
F95K	GGGGAATGGCTTTATCTGCCTTAAGTG	94572	94598	F	F95K + R102K PCR
R102K	CTCCTCTATCTCTCCCCCTATAAGTG	101792	101767	R	F95K + R102K PCR
F101K	GAAGTGCCAGAAGAAAATAAGGACTTGCC	101089	101117	F	F101K + R108R PCR
R108R	CTTAGTCGTCATTAGTGGCTTGGTTGTG	108317	108290	R	F101K + R108R PCR
F108K	GTCACCACTCTCCACAATGTAGTAGTC	108078	108104	F	F108K + R115K PCR
R115K	CGTATCAGAGCTACTATTAGAAGAGGAGG	115332	115304	R	F108K + R115K PCR
F115K	GTACAGATGCTAGTAGTTCGTGGTCTTC	115044	115071	F	F115K +R122K PCR
R122K	CAGAGCAACCAATTAATGAACGCACAGAG	122317	122289	R	F115K +R122K PCR
F122K	CTTCATCCGTCATGTGCTCTACTAATGTG	121956	121984	F	F122K + R130K
R130K	CAATTGATGCAATCATCCGCTCAGGAC	130004	129978	R	F122K + R130K
E5M4rp1	GCATGACGAGACATCTCATGCTC	128013	128035	F	B8M4p3' +E5M4rp1 PCR
B8M4p3'	GCGAAGTAGGGGTTTCAGAGTGTG	131769	131746	R	B8M4p3' +E5M4rp1 PCR
F130K	CACCTACGATTGTAGTTACCCAACCATG	129747	129774	F	F130K + R137K PCR
R137K	CTCTTAACATGCGAACCAAGGTAATCGG	136966	136939	R	F130K + R137K PCR
F137K	GCATACCCTCTTCAATAGCTCGTTGAG	136301	136327	F	F137K + R1K PCR
R1K	GAAATTAGCTCCTCTTCTTCACCGTCC	1017	991	R	F137K + R1K PCR

a “Forward” and “Reverse” are abbreviated as “F” and “R”, respectively.
