# Supplementary material for: Improved Adsorption of an Enterococcus faecalis Bacteriophage ΦEF24C with a Spontaneous Point Mutation
Source: PLoS One. 2011 Oct 25;6(10):e26648. doi: 10.1371/journal.pone.0026648 (PMC3201976; doi:10.1371/journal.pone.0026648)
Supplement: Table S2 — List of genome sequencing primers. (PDF) [file pone.0026648.s006.pdf]

Table S2. List of genome sequencing primers.

No	PCR Fragment	Primer	Location		Direction	Sequence
			Start	End		
1	F1K + R8K PCR	orf34 R1	1524	1505	R	CCTATGCCCGTTTAACAGAG
2	F1K + R8K PCR	Aftorf34 R1-p14	1304	1322	F	CACACCGACCTTGGCAACC
3	F1K + R8K PCR	phiEF24C Lyt F2	1985	2002	F	GTATGACCGTGTACCTGC
4	F1K + R8K PCR	Aftorf34 R1-p16	2316	2332	F	GACAGATGGTACAACGG
5	F1K + R8K PCR	phiEF24C Lyt F5'	3217	3238	F	GGGTGTCAACTCAATTAGCACC
6	F1K + R8K PCR	phiEF24C Lyt F6	3744	3762	F	GCTAAGGAACTACGTAAGG
7	F1K + R8K PCR	phiEF24C Lyt F7	4400	4418	F	GCTACTGATGAGGACTACG
8	F1K + R8K PCR	phiEF24C Lyt F8	4942	4960	F	CAGTATGAGAGAGGGGCTG
9	F1K + R8K PCR	phiEF24C Lyt F9	5481	5498	F	GGTATCCCTATCGACACC
10	F1K + R8K PCR	phiEF24C Lyt F10	5961	5980	F	CCTACACCAAGCAAACCAAG
11	F1K + R8K PCR	LytNp2	6759	6778	F	CGCACCAGTAGCACCTGAAC
12	F1K + R8K PCR	LytN p2-2	7431	7454	F	GGTAACCCTGTAATTTATGATTAC
13	F8K + R15K PCR	F8K	7924	7949	F	GCCCGCAAGAAACTCTGAAACAGGTG
14	F8K + R15K PCR	/kpn1 8k-M4p3	8514	8532	F	GGAGAAGCTCACTAGCCGG
15	F8K + R15K PCR	/kpn1 8k-M4p4	9091	9110	F	GCTATCTGGTGGAGATGTAG
16	F8K + R15K PCR	/kpn1 8k-M4p5	9648	9667	F	GGTGCCCCAACTAGCAATTG
17	F8K + R15K PCR	/kpn1 8k-M4p6	10202	10219	F	GGGGTAAGAAATAGTGGC
18	F8K + R15K PCR	/kpn1 8k-M4p7	10773	10790	F	GAAGCGGAGTCTGTTGAG
19	F8K + R15K PCR	/kpn1 8k-M4p8	11343	11361	F	GCTTCACTACAGGATATGG
20	F8K + R15K PCR	/kpn1 8k-M4p9	11906	11922	F	GGTACACCAACAGATGC
21	F8K + R15K PCR	/kpn1 8k-M4p10	12474	12491	F	CGGACGTATTCGTTGGAG
22	F8K + R15K PCR	/kpn1 8k-M4p11	13033	13049	F	GAGGAAGACTAAGGAGG
23	F8K + R15K PCR	/kpn1 8k M4rF	13518	13541	F	GTACCCAACATCCCTCATGCAGAC
24	F8K + R15K PCR	/kpn1 8k M4rFp1	14000	14018	F	GGTGGAGCAGGTGAAACAG
25	F8K + R15K PCR	/kpn1 8k M4rFp2	14453	14463	F	GGTAAAGACGGCGTATC
26	F8K + R15K PCR	/kpn1 8k M4rFp3	14943	14962	F	GTTGCTCTACGTAATGGGTC
27	F8K + R15K PCR	E4M4rp4	14113	14094	R	CCACAAAACGTCAATACCCC

Continued on following page

Table S2 - Continued.

No	PCR Fragment	Primer	Location		Direction	Sequence
			Start	End		
28	F15K + R21K PCR	E4M4rp1	15717	15697	R	CTTCTTCGCTACTACCGAGAG
29	F15K + R21K PCR	E4M4rp2	15162	15143	R	GAACTGCTCTCAGCTCATCG
30	F15K + R21K PCR	E4RVp1	17580	17563	R	CGCATTGGGTCACCATTG
31	F15K + R21K PCR	E4RVp2	17050	17031	R	GTTGGCTTGAGCTTCACTAC
32	F15K + R21K PCR	E4RVp3	16496	16480	R	CAGCATCCTCGTGTTGG
33	F15K + R21K PCR	E4RVp4	16017	15999	R	GGTATCCTTTGTAGACACC
34	F15K + R21K PCR	E3M4rF4	21055	21037	R	CGATAGCCTCCTGAATATC
35	F15K + R21K PCR	E3M4rF4'	20575	20557	R	GCCACGCAGGGTCACTCGG
36	F15K + R21K PCR	E3M4rF5	19824	19805	R	CTTCAACACGTTTCACAACG
37	F15K + R21K PCR	E3M4rF7	19157	19138	R	CCCTAACAAACCTTACACGC
38	F15K + R21K PCR	E3M4rF8	18351	18335	R	CCTGTGTGGACTGTTTG
39	F15K + R21K PCR	R21K	21618	21590	R	CTCTGTTTAGTTCCTGTTGTGCCCTAGAG
40	F20K + R26K PCR	F20K	20361	20388	F	GTAGAGGTGCCAGAAGTACTAGCTGATG
41	F20K + R26K PCR	R26K	25957	25931	R	CTGTTTAGCTGTTGAGCCATCGCTCTC
42	F20K + R26K PCR	E5RVrp6	20482	20500	F	GGTGGAATAAAAGCCCTCG
43	F20K + R26K PCR	E5RVrp7	21180	21198	F	GTTGAGGCAATGACTGGGG
44	F20K + R26K PCR	E5RVrp8	22011	22028	F	CGTGGTGTTCTAGCTGAG
45	F20K + R26K PCR	E5RVrp9	22800	22818	F	CAATCAGTTCCCGGTGACG
46	F20K + R26K PCR	E3M4p1	23595	23612	F	GCTAAGCTAGGGGCAATG
47	F20K + R26K PCR	E1RVp1	24973	24954	R	GCTTACCTTCTCTGCTTCAC
48	F20K + R26K PCR	E1RVp2	24432	24414	R	CTGGGCTAGTTATGTGTCC
49	F20K + R26K PCR	E1RVp3'	23896	23879	R	GCAGACTCTTCTGCGTTG
50	F20K + R26K PCR	A2RVr1	25950	25931	R	GCTGTTGAGCCATCGCTCTC
51	F25K + R31K PCR	F25K	24946	24973	F	CAATAACGGTGAAGCAGAGAAGGTAAGC
52	F25K + R31K PCR	R26K	25957	25931	R	CTGTTTAGCTGTTGAGCCATCGCTCTC
53	F25K + R31K PCR	outH1RVp2	29471	29452	R	CTGTCAACCTCGTAGGTTTC
54	F25K + R31K PCR	outH1RVp3	28915	28899	R	CTCTGCCATCTGGTGTC
55	F25K + R31K PCR	outH1RVp4	28379	28361	R	GCTATCGGGTTAACCTGTC
56	F25K + R31K PCR	outH1RVp5	27813	27795	R	GACTTCTGACCTCTAGTAG
57	F25K + R31K PCR	outH1RVp6	27238	27222	R	GTAAACCCCCGCACTAC
58	F25K + R31K PCR	outH1RVp7	26715	26704	R	CCTCCGGAGAAGGTCCTCAG
59	F25K + R31K PCR	A2RVp7	28980	28996	F	CAACAATGAGGGGTTCG
60	F25K + R31K PCR	A2RVp8	29543	29561	F	CTAGCTCGGTAGACGGAGC
61	F25K + R31K PCR	Pst1C2H1RVrF	31125	31102	R	GAACCCTACATAGTTGTACCCACC
62	F25K + R31K PCR	Pst1C2H1RVrF1	30605	30585	R	GTAGCCTTTGTACCTGCCTCG

Continued on following page

Table S2 - Continued.

No	PCR Fragment	Primer	Location		Direction	Sequence
			Start	End		
63	F30K + R36K PCR	Pst1C2H1RVrF	31125	31102	R	GAACCCTACATAGTTGTACCCACC
64	F30K + R36K PCR	Pst1C2H1RVrF1	30605	30585	R	GTAGCCTTTGTACCTGCCTCG
65	F30K + R36K PCR	R31K	31693	31665	R	GACCCTGTAAATCGTTAGCCCAAGCAGTG
66	F30K + R36K PCR	H1RVr	33408	33386	R	GTAGCCTGAAAACTCTTGGGGAG
67	F30K + R36K PCR	H1RVrp1	32874	32855	R	CGCCCCGTCTTCCTTGTTAG
68	F30K + R36K PCR	H1RVrp2	32338	32320	R	CATCCCAGAAGTCTAACCC
69	F30K + R36K PCR	C2H1M5rRp6'	33947	33924	R	ACTCTCCACTCTTTAGCTAGTAAC
70	F30K + R36K PCR	Pst1c2H1M5rRp5	34590	34571	R	CGACATTGTGTTCCCTGCTG
71	F30K + R36K PCR	H1M5rp1	34231	34248	F	GCCCATTGGACGTACTTG
72	F30K + R36K PCR	F35K	34981	35006	F	GGAGCACCTGCTGTAGAATTAGGAGC
73	F35K + R41K PCR	F35K	34981	35006	F	GGAGCACCTGCTGTAGAATTAGGAGC
74	F35K + R41K PCR	H1M5rp4	35796	35814	F	GCTTTGGAAAGGGGTTGTC
75	F35K + R41K PCR	H1M5rp5	36346	36365	F	GGGACTGTGAAGAGACCTAC
76	F35K + R41K PCR	H1M5rp6	36853	36875	F	GGAGGTGATATTTCTATGTACCC
77	F35K + R41K PCR	Sg2to3p1	37526	37543	F	GCACCAGAAGGGCTATCC
78	F35K + R41K PCR	Sg2to3'	37362	37386	F	CAGGATTCAGGGTAGACAGCAGAGG
79	F35K + R41K PCR	Sg2to3p2	38301	38319	F	CTATGGATGTCGCTCTAGG
80	F35K + R41K PCR	Sg2to3p3	39036	39057	F	CAAAACGATAACTCGGGGGCTC
81	F35K + R41K PCR	Sg2to3p4	39515	39540	F	CATACCTAAAGGAAGAATCAATCGGG
82	F35K + R41K PCR	Sg2to3p5	40367	40386	F	CCGTAAAGATGGTTGGGACG
83	F40K + gp41R PCR	F40K	39821	39848	F	GGACTACAGACCTGCAGGCATTAAACTG
84	F40K + gp41R PCR	Sg2to3p5	40367	40386	F	CCGTAAAGATGGTTGGGACG
85	F40K + gp41R PCR	Sg2to3p6	41190	41213	F	GCATTGAACACCCTATTAGGGAAC
86	F40K + gp41R PCR	Sg2to3p7	41920	41942	F	CGCTAATTCCAGATGAGGACGGC
87	F40K + gp41R PCR	Sg2to3p8	42615	42636	F	GTGGTGTTCCTAGCCATTTCTC
88	F40K + gp41R PCR	Sg2to3p9	43335	43355	F	CTGGTGAAGGCGATGCTAGGG
89	F40K + gp41R PCR	Sg2to3p10	44023	44045	F	CCCTTCTGCAGATAACTGGATTG
90	F40K + gp41R PCR	Sg2to3p11	44711	44732	F	CGTGGAATATCAGAAGGTGGGC
91	F40K + gp41R PCR	gp41R	45662	45638	R	GTCTTCTTACTCTTGGACGGAGGAA
92	Sg2to3p11 + R46K	Sg2to3p11	44711	44732	F	CGTGGAATATCAGAAGGTGGGC
93	Sg2to3p11 + R46K	gp42F1	45777	45801	F	cacagacaaacaagctcaggtgcgc
94	Sg2to3p11 + R46K	gp42R1	45888	45864	R	gttctttctctagctctgttctctc

Continued on following page

Table S2 - Continued.

No	PCR Fragment	Primer	Location		Direction	Sequence
			Start	End		
95	F46K + R53K PCR	F46K	46165	46190	F	CTAAGGTGGGTATGTCAAGGAGCATG
96	F46K + R53K PCR	F2RVp2	46412	46429	F	GATACGTTCCCCACAGGG
97	F46K + R53K PCR	F2M4r1	46817	46840	F	GTAGGTGCTTTTGGTGCAGGTAAG
98	F46K + R53K PCR	F2M4r2	47293	47312	F	CGCTCAGTATAGAATGGCAC
99	F46K + R53K PCR	F2M4r3	47840	47857	F	GGTCGGGGCTTACGTAAG
100	F46K + R53K PCR	F2M4r4	48419	48438	F	GCGGAGATAGCTGAGGCACG
101	F46K + R53K PCR	F2M4r5	49226	49241	F	CGTGGGGCTAACTTCAACAC
102	F46K + R53K PCR	F2M4r6	49924	49944	F	GTCTTTGAGGTAGTTAGCGAC
103	F46K + R53K PCR	F2M4r7	50259	50280	F	GCTAGCTCGTGGAGAAGTCGGG
104	F46K + R53K PCR	F2M4r8	50879	50900	F	CATCAGAGGAGTTTAGTAACGG
105	F46K + R53K PCR	F2M4r9	51556	51576	F	GTTATGGGGAACAGGTCGAAG
106	F46K + R53K PCR	F2M4r10	52189	52211	F	GTTTGAGAGAGGCAATGGAAGTG
107	F46K + R53K PCR	F2M4r11	52846	52867	F	GGTGTTTACCGATATGCACAGG
108	F53K + R60K PCR	F53K	53435	53411	F	GCTACCTATGTGGTGCTTTATTGAGCC
109	F53K + R60K PCR	E7RVp1	54796	54779	R	GTAGCCTTTTCCTCTCTG
110	F53K + R60K PCR	E7M4p1	54061	54079	F	GCAATTGGTTGTGAGAACG
111	F53K + R60K PCR	E7M4p2	54779	54796	F	CAGAGAGGAAAAGGCTAC
112	F53K + R60K PCR	E7M4p3	55305	55333	F	CTAAGTCAGAACCTGAGAGAGCCAGAAGC
113	F53K + R60K PCR	E7M4p3p1	56011	56032	F	CTACGCTGTACAGACGATAAAG
114	F53K + R60K PCR	E7M4p3p2	56816	56837	F	GTTCGTTGGGACTACGGAAACC
115	F53K + R60K PCR	E7M4p3p3	57580	57602	F	GAAAAACACCAGTGCAGTGTAAC
116	F53K + R60K PCR	E7M4p3p4	58366	58384	F	CGAAACAAACGTATCGGGG
117	F53K + R60K PCR	E7M4p3p5	59171	59192	F	GTAGACGACTACAAGGACACTC
118	F53K + R60K PCR	E9M5R7	57708	57689	R	GTCTGCCATCCCTACATAAG
119	F53K + R60K PCR	E9M5R8	56977	56957	R	GGGAATAACTACTCCATCGTC

Continued on following page

Table S2 - Continued.

No	PCR Fragment	Primer	Location		Direction	Sequence
			Start	End		
120	F60K + R68K PCR	F60K	60002	60030	F	CAGAGATGCTCAGCTAATAGAAATACCAG
121	F60K + R68K PCR	E3M4p3p7	60657	60677	F	GCTATGAGACAGAGAGATTG
122	F60K + R68K PCR	E3M4p3p8	61438	61459	F	CTAGTGTCATAGAGCAACAGAG
123	F60K + R68K PCR	E7M4p3p9	62174	62193	F	CGTGGTGACTTATTAGGGGC
124	F60K + R68K PCR	E9M5in	62674	62698	F	GCTTATTGGTAAACCTACTAAGTCC
125	F60K + R68K PCR	E9M4p1	63088	63107	F	CTACAAAGAGGGGCTTAAAG
126	F60K + R68K PCR	E9M4p2	63567	63585	F	GTAGTAAGGTTGGAGACCC
127	F60K + R68K PCR	E9M4p3	64094	64111	F	CAAGGAACTGGTGCCTAC
128	F60K + R68K PCR	E9M4p6	64763	64788	F	GTGAGAACAGCATTTTTAGAGCAACC
129	F60K + R68K PCR	E9RVrFp1	65408	65425	F	GCAGTACCGGGAGAAAAC
130	F60K + R68K PCR	E9RVrFp2	65918	65936	F	GCATTACCACAAGGTTGGG
131	F60K + R68K PCR	E9RVrFp3	66487	66506	F	CACACCTAGTGTAGACGACC
132	F60K + R68K PCR	E9RVrFp4	67013	67030	F	GAACGTGTAGCAGAAGAG
133	F60K + R68K PCR	F1M4p1	67786	67803	F	GGAACAACCTAACCAAGC
134	F60K + R68K PCR	17RVRp1	67923	67946	F	GCGCAAAGGACTAGCACCTAACCC
135	F68K + R76K PCR	F1RVp2	68631	68612	R	GCGGTATCTCGGAACCGTCC
136	F68K + R76K PCR	F1M4p2'	68792	68811	F	GGAACAGTAAGGGATTCTCG
137	F68K + R76K PCR	F1M4p3	69373	69391	F	GATGGGTACACTGGAACCG
138	F68K + R76K PCR	F1RVr1	70132	70151	F	GCAAGCGGTGACGTTGTAGC
139	F68K + R76K PCR	F1RVr3	70958	70980	F	CATTAGTGGGTTTGTAGAGGCGG
140	F68K + R76K PCR	F1RVr4	71830	71849	F	GCATGTTAACGAAGTAGCCC
141	F68K + R76K PCR	F1RVr5	72505	72526	F	CAACGTGAGGGAGCTTATCTTG
142	F68K + R76K PCR	F1RVr6	73233	73255	F	CATGGGGCATATCCATACAAC
143	F68K + R76K PCR	F1RVr8	74620	74632	F	CGAGGGCTCATTGGCATAG
144	F68K + R76K PCR	F1RVr9p1	75492	75520	F	GCATTGGTAAGGAACCTGTAGGTAAGCTG
145	F68K + R76K PCR	F1RVp1	69084	69068	R	GGTAAAGGTTCCCAACC
146	F68K + R76K PCR	F1RVp2	68631	68612	R	GCGGTATCTCGGAACCGTCC
147	F68K + R76K PCR	SG2cl4SLr	74997	74973	R	CCCACCAGAACGGCTGTTTGTCTTC
148	F68K + R76K PCR	SG2cl4SLr1	74543	74520	R	CCTATATCCCCGAATATTCTTTGC

Continued on following page

Table S2 - Continued.

No	PCR Fragment	Primer	Location		Direction	Sequence
			Start	End		
149	F1RVr5 + F1RVr9p3R5 PCR	F1RVr6	73233	73255	F	CATGGGGCATATCCATACAAC
150	F1RVr5 + F1RVr9p3R5 PCR	F1RVr7	73914	73933	F	CATTAATCAGACGTGGGGTC
151	F1RVr5 + F1RVr9p3R5 PCR	F1RVr8	74620	74632	F	CGAGGGCTCATTGGCATAG
152	F1RVr5 + F1RVr9p3R5 PCR	F1RVr9	74826	74855	F	GTTAGGTGAGTATAAACAACCTAGCCGTTC
153	F1RVr5 + F1RVr9p3R5 PCR	F1RVr9p1	75492	75520	F	GCATTGGTAAGGAACCTGTAGGTAAGCTG
154	F1RVRVr9 + F1RVr9p3R PCR	F1RVr9p1	75492	75520	F	GCATTGGTAAGGAACCTGTAGGTAAGCTG
155	F1RVRVr9 + F1RVr9p3R PCR	F1RVr9p1-1	76277	76298	F	CAGACCAGTAGGAGAAAGACAG
156	F1RVRVr9 + F1RVr9p3R PCR	F1RVr9p2	76617	76644	F	GGTGTGCACACTTACACCGAAGCACTAG
157	F1RVRVr9 + F1RVr9p3R PCR	F1RVr9p2-1	77375	77395	F	GCCACAAGCTCCCAAAGTTTC
158	F1RVRVr9 + F1RVr9p3R PCR	F1RVr9p2-2	78222	78241	F	GCTGTGCTGATTAAGAATGG
159	F1RVRVr9 + F1RVr9p3R PCR	F1RVr9p2-3	79116	79136	F	GCTCTTAAGACTGCTCATGAC
160	F1RVRVr9 + F1RVr9p3R PCR	F1RVr9p2-4	79906	79926	F	CTAAGGTAGACGAGGCGTTAG
161	F1RVRVr9 + F1RVr9p3R PCR	F1RVr9p2-5	80683	80703	F	GGTGTTGTACCTGTTACGCAG
162	F1RVRVr9 + F1RVr9p3R PCR	F1RVr9p3	81324	81348	F	GCGTAAGACACAAGGCGCAGAACCT
163	F1RVr9p3 + F1RVr9p4SR1 PCR	F1RVr9p4KR1	82537	82519	R	GTGGGTGATTCTCTACACG
164	F1RVr9p3 + F1RVr9p4SR1 PCR	F1RVr9p4KR2	81711	81691	R	CCAAGCTTGCTTCATGTAGTC
165	F1RVr9p3 + F1RVr9p4SR1 PCR	F1RVr9p4	81809	81833	F	CAGACCATATTGTTACCTTGTATGC
166	F1RVr9p3 + F1RVr9p4SR1 PCR	F1RVr9p4K1	82485	82506	F	CTATGCAGTACGTTCAGGAGAC
167	F1RVr9p3 + F1RVr9p4SR1 PCR	F1RVr9p4K2	83353	83372	F	CGAGTGCCACGTAGAGGACG
168	F1RVr9p3 + F1RVr9p4SR1 PCR	F1RVr9p4K3	83937	83956	F	GTAGATGGCGGTTTGGTAGC
169	F1RVr9p3 + F1RVr9p4SR1 PCR	F1RVr9p4K4	84734	84755	F	CTGGTAAGGGAAGCTAAACTAC
170	F1RVr9p3 + F1RVr9p4SR1 PCR	F1RVr9p4K5	85600	85619	F	CTTATGGAGACTACCATGGG
171	F1RVr9p3 + F1RVr9p4SR1 PCR	F1RVr9p4K6	86389	86408	F	GAACGAGGTCAAGTAGTTAC
172	F1RVr9p3 + F1RVr9p4SR1 PCR	F1RVr9p4K7	86932	86952	F	GCTACACTAACAGTTCCTGTG

Continued on following page

Table S2 - Continued.

No	PCR Fragment	Primer	Location		Direction	Sequence
			Start	End		
173	F87K + R95K PCR	F87K	87199	87224	F	GTAGCAGGCTACACAGAGTCTACTTC
174	F87K + R95K PCR	F1RVr9p5	87333	87362	F	CGGTGGTGTAACATTAACAGTTGTAGTGGC
175	F87K + R95K PCR	F1RVr9p6	87903	87927	F	GCACCCTCTTAAGAAGTTACAAGTA
176	F87K + R95K PCR	F1RVr9p7	88532	88558	F	GCCATCTTACTATCAGACGTAGAGGAC
177	F87K + R95K PCR	F1RVr9p8	88803	88827	F	GGCTAGAGGGTACTCGTTGTGGACG
178	F87K + R95K PCR	F1RVr9p9	89530	89557	F	GGTAATTGGGGTTGTCGCTACATGGTTG
179	F87K + R95K PCR	F1RVr9p9-1	90375	90393	F	GCTGAGGAGGCAAGAGCAC
180	F87K + R95K PCR	F1RVr9p9-2	91224	91246	F	GGTTGAGCTTACACTAGTAGAAG
181	F87K + R95K PCR	F1RVr9p9-3	92061	92081	F	CTCTAGTATGGCTAGCTTTGG
182	F87K + R95K PCR	F1RVr9p9-4	92861	92882	F	GTAGGGGACTTCATTTTATGGG
183	F87K + R95K PCR	F1RVr9p9-5	93646	93666	F	GTGTGTAGCGATTGCTATTCC
184	F87K + R95K PCR	F1RVr9p10	94097	94123	F	CCCAAGTAGAGGGTCTTAAACTAGGTG
185	F87K + R95K PCR	F1RVr9p9R	94201	94173	R	CCTCATCAACAACAAACCCTTGCTTGTTC
186	F87K + R95K PCR	F1RVr9p9R1	93317	93296	R	CTAAACTCAACCTAGCATCTGG
187	F87K + R95K PCR	F1RVr9p9R2	92496	92474	R	CTCTCTGTTGCTATTGAAGCTAG
188	F87K + R95K PCR	F1RVr9p9R3	91716	91698	R	GCCATGGTTTACCTTAGGC
189	F87K + R95K PCR	F1RVr9p9R4	90900	90881	R	GCAAACAGGCACTCTGCCTC
190	F87K + R95K PCR	F1RVr9p9R5	90058	90040	R	GCTCATTGGGATGTGGCGG
191	F95K + R102K PCR	F95K	94572	94598	F	GGGGAATGGCTTTATCTGCCTTAAGTG
192	F95K + R102K PCR	F1RVr9p10-1	94711	94738	F	GGGTAAACTGCTAGAAGTCTCTGATAAC
193	F95K + R102K PCR	F1RVr9p10R2	96239	96219	R	GTGGGTTGTAATAGGTAGCAC
194	F95K + R102K PCR	F1RVr9p10R3	95527	95504	R	GAAAGCTGTCCCCAACCGTCACAC
195	F95K + R102K PCR	F1RVr9p10-2	95504	95527	F	GTGTGACGGTTGGGGACAGCTTTC
196	F95K + R102K PCR	F1RVr9p9R	94201	94173	R	CCTCATCAACAACAAACCCTTGCTTGTTC
197	F95K + R102K PCR	F1RVr9p10-4	97046	97070	F	CTAATGGGGTTGCTTGTTAGCGTTG
198	F95K + R102K PCR	F1RVr9p11	97429	97455	F	GAGTGAGTGGCTACTTAGGGACTTAGG
199	F95K + R102K PCR	F1RVr9p11-1	98195	98216	F	GACTTCCTCCCCTTTACATAGAC
200	F95K + R102K PCR	F1RVr9p11-1'	98249	98271	F	GCACTAAACATTGCAGAGAATAG
201	F95K + R102K PCR	B8M4p3p12	100326	100299	R	GGTATTTTCCCGCTAACTCTTTACTAGC
202	F95K + R102K PCR	B8M4p3p12-1	99493	99469	R	CTAATGTTTCCATGTTTAGCACTTC
203	F95K + R102K PCR	F1RVr9p11-2	99555	99575	F	CGTTAAAAACGGGGTTGACAC
204	F95K + R102K PCR	B8M4p3p11R	100299	100321	F	GCTAGTAAAGAGTTAGCGGGAAAT
205	F95K + R102K PCR	B8M4p3p11R1	101079	101101	F	GAAACTAGAAGAAGTGCCAGAAG
206	F95K + R102K PCR	F1RVr9p9R	94201	94173	R	CCTCATCAACAACAAACCCTTGCTTGTTC
207	F95K + R102K PCR	B8M4p3p12	100326	100299	R	GGTATTTTCCCGCTAACTCTTTACTAGC
208	F95K + R102K PCR	B8M4p3p12-1	99493	99469	R	CTAATGTTTCCATGTTTAGCACTTC
209	F95K + R102K PCR	F1RVr9p10R2	96239	96219	R	GTGGGTTGTAATAGGTAGCAC
210	F95K + R102K PCR	F1RVr9p10R3	95527	95504	R	GAAAGCTGTCCCCAACCGTCACAC

Continued on following page

Table S2 - Continued.

No	PCR Fragment	Primer	Location		Direction	Sequence
			Start	End		
211	F101K + R108R PCR	B8M4p3p11R1	101079	101101	F	GAAACTAGAAGAAGTGCCAGAAG
212	F101K + R108R PCR	B8M4p3p11R1.1	101767	101787	F	CACTTATAGGGGGAGAGATAG
213	F101K + R108R PCR	B8M4p3p11R2	101970	101988	F	GAAAGGGGGAGGGAAAAGG
214	F101K + R108R PCR	B8M4p3p11R3	102785	102804	F	GCCTATTTTAGAGGGCTACC
215	F101K + R108R PCR	B8M4p3p11R4	103568	103585	F	GGAGGGGGCTACAATGGAC
216	F101K + R108R PCR	B8M4p3p11R5	104400	104418	F	CGGGGGCTACTAACATGAC
217	F101K + R108R PCR	B8M4p3p10R	104862	104886	F	GTTAAAGTACCGAGTGAGTTGCTAA
218	F101K + R108R PCR	B8M4p3p10R1	105656	105673	F	GGGAACGACATCGACAAC
219	F101K + R108R PCR	B8M4p3p10R2	106514	106537	F	CAGACACGACTATTGCATTCTTTG
220	F101K + R108R PCR	B8M4p3p9R3	107369	107388	F	GGAAAAGGGCACGGCACTTG
221	F101K + R108R PCR	B8M4p3p8R	107938	107962	F	CCCATATAAAAAGACCCCTCCCCGT
222	F101K + R108R PCR	B8M4p3p10	107626	107602	R	CAAGCCCAACCCGCTTACTGCTTGT
223	F101K + R108R PCR	B8M4p3p9-2	106771	106749	R	CATAGACTTGCTGTAATTCCTCG
224	F101K + R108R PCR	B8M4p3p9-3	105900	105882	R	CCCCTCTGACTCTATAACC
225	F101K + R108R PCR	B8M4p3p9-4	105341	105323	R	GTTCGGTCCTCGTTCATTG
226	F101K + R108R PCR	B8M4p3p11	105062	105038	R	GCGACTAACGTTAACACCTAACAAC
227	F101K + R108R PCR	B8M4p3p11-1	104645	104621	R	CGTGTTTAGCTTCTATAACCGTGTC
228	F101K + R108R PCR	B8M4p3p11-2	103889	103865	R	CGTCTAGTGTGCTGTATTCCTCTTC
229	F101K + R108R PCR	B8M4p3p11-3	103078	103054	R	CGGGACTCTGGTCTACTATATCTCC
230	F101K + R108R PCR	B8M4p3p11-4	102242	102221	R	GATATAAGCGCCCCCTATATAG
231	F101K + R108R PCR	B8M4p3p11-5	101365	101344	R	CTAGGTACTTTCTAACCTCCTC
232	F108K + R115K PCR	F108K	108078	108104	F	GTCACCACTCTCCACAATGTAGTAGTC
233	F108K + R115K PCR	B8M4p3p8R1	108751	108771	F	GTCACACTAGTTACTGTTGCC
234	F108K + R115K PCR	B8M4p3p8R2	109612	109631	F	CGTGTAGGTACTGTGTACTC
235	F108K + R115K PCR	B8M4p3p8R3	110347	110368	F	GTATAGTCTTCCTCACCTGTTG
236	F108K + R115K PCR	B8M4p3p7R1	111759	111782	F	GGGTAACTTCCTTTGTCAATCATG
237	F108K + R115K PCR	B8M4p3p7R2	112561	112579	F	GTGGGTAGCTGTTGCCATC
238	F108K + R115K PCR	B8M4p3p7R3	113408	113430	F	CTGCCTCTACTGCAGTCTTATAC
239	F108K + R115K PCR	B8M4p3p6	114475	114446	R	GATTAAGCTAAGAGAGCGGGGTATATTTGG
240	F108K + R115K PCR	B8M4p3p6-1	113765	113743	R	CACCCATTATGCACAGAAAGACG
241	F108K + R115K PCR	B8M4p3p7	113569	113554	R	GTCACGTAGAGGACGAGGAATAGTG
242	F108K + R115K PCR	B8M4p3p7R4	114133	114151	F	CCTTCTCTGTCATTGCCTG
243	F108K + R115K PCR	B8M4p3p5R	114296	114325	F	CTGAATACCCGTTCTAGGTCATTCAATGAG
244	F108K + R115K PCR	B8M4p3p5R1	115101	115120	F	CTCCAAGCTCTACCACAAAG
245	F108K + R115K PCR	110831F	110830	110852	F	CCCCTGAACTCCCGAGACGTGGC
246	F108K + R115K PCR	111955R	111954	111929	R	CTCAAGTAGAGTGTGACGGTACATTG

Continued on following page

Table S2 - Continued.

No	PCR Fragment	Primer	Location Start	Location End	Direction	Sequence
247	F115K +R122K PCR	F115K	115044	115071	F	GTACAGATGCTAGTAGTTCGTGGTCTTC
248	F115K +R122K PCR	B8M4p3p5R2	115892	115912	F	CCCTCAATAGGTGCAGTGAAC
249	F115K +R122K PCR	B8M4p3p5R3	116761	116780	F	GCAGATTGGAGGAGCTCATC
250	F115K +R122K PCR	B8M4p3p5R4	117621	117643	F	CGTACTCTGCTTTCTCAACAAAC
251	F115K +R122K PCR	B8M4p3p3R	118338	118362	F	CTTCCATTGTCTTGCCCTCCCTATC
252	F115K +R122K PCR	B8M4p3p3R1	119156	119176	F	CCCTCACTACTTGCTACCTCC
253	F115K +R122K PCR	B8M4p3p3R2	122021	120042	F	CTGCTAGCCAGTAAGTAGCTAG
254	F115K +R122K PCR	B8M4p3p3R3	120848	120870	F	CTGGTGAGTCTACCCCTACATTC
255	F115K +R122K PCR	B8M4p3p2-4	121766	121745	R	GAGGGAGCACCTAACGAATATG
256	F115K +R122K PCR	B8M4p3p3R4	121616	121638	F	GCGATATAGTCACTCTGTAAGGC
257	F122K + R130K	B8M4p3p2-3	122556	122542	R	GCCAATACTCATTGAGGAATGG
258	F122K + R130K	B8M4p3p3R5	122430	122450	F	GTAGCCCACCGTTTCGTTGTG
259	F122K + R130K	B8M4p3p3R6	123240	123263	F	GCAGTCATCACTTAAAGCATTATC
260	F122K + R130K	B8M4p3p3R7	124100	124123	F	CCCTACTATTGTGATTCTGTCAAC
261	F122K + R130K	B8M4p3p2R1	124872	124896	F	CTTGCTCACTGGTTGCTCTTACTGG
262	F122K + R130K	B8M4p3-7	125901	125880	R	GAGGTAGAAGTACCAAAACAGG
263	F122K + R130K	B8M4p3p2R2	125762	125786	F	GCGAAAAACCATTTACTAAACTCTG
264	F122K + R130K	B8M4p3p2R3	126635	126655	F	CTACGTCATCCTCTCATTAGC
265	F122K + R130K	B8M4p3p2R4	127497	127516	F	CTTACTCATAGGCTACCTCC
266	F122K + R130K	E5M4rp1	128013	128038	F	GCATGACGAGACATCTCATGCTC
267	F122K + R130K	B8M4p3p2R5	128311	128332	F	CCCTACAGTGTTTGCCATAACC
268	F122K + R130K	B8M4p3'-1	130901	130882	R	CCCAACAACAAGTGCAGACG
269	F122K + R130K	B8M4p3-2	130043	130023	R	CGGTAGGTGTGCCTGACGTTG
270	F122K + R130K	R130K	130004	129978	R	CAATTGATGCAATCATCCGCTCAGGAC
271	B8M4p3' +E5M4rp1 PCR	B8M4p3p2R6	129122	129145	F	CCATGCTCTTTTTAGCTGTTCTTC
272	B8M4p3' +E5M4rp1 PCR	B8M4p3'-1	130901	130882	R	CCCAACAACAAGTGCAGACG
273	B8M4p3' +E5M4rp1 PCR	B8M4p3-2	130043	130023	R	CGGTAGGTGTGCCTGACGTTG
274	B8M4p3' +E5M4rp1 PCR	R130K	130004	129978	R	CAATTGATGCAATCATCCGCTCAGGAC

Continued on following page

Table S2 - Continued.

No	PCR Fragment	Primer	Location		Direction	Sequence
			Start	End		
275	F130K + R137K PCR	F130K	129747	129774	F	CACCTACGATTGTAGTTACCCAACCATG
276	F130K + R137K PCR	B8M4p3p2R8	130733	130756	F	CTATACTAAACTAGCTAGCTTGTC
277	F130K + R137K PCR	B8M4p3p2R9	131494	131515	F	CCGATTCTGATTGAACTGTGTC
278	F130K + R137K PCR	B8RVp1	132259	132278	F	CTTCTGCCACCTTACTAGAG
279	F130K + R137K PCR	B8RVp2	133006	133024	F	CCCCAGACTCAGCTAGCTC
280	F130K + R137K PCR	Aftorf34 R5	133787	133810	F	GAATGCTCTAACAGCTTCTTTATC
281	F130K + R137K PCR	Aftorf34R5-3	134474	134496	F	CCTTCATCGTTCGTGTATGTTCC
282	F130K + R137K PCR	B8RVp3p1-2	135788	135806	F	CAACAGGCTGTAGTCCACC
283	F130K + R137K PCR	Aftorf34 R2	136531	136550	F	GTTTGCGCAGGCAACTTATC
284	F130K + R137K PCR	B8M4p3'-1	130901	130882	R	CCCAACAACAAGTGCAGACG
285	F130K + R137K PCR	Aftorf34F2'-1	136041	136022	R	CGACTACCCTGATGCAGACG
286	F130K + R137K PCR	Sg1Rp2	135815	135791	R	caatttgaaggtggactacagcctg
287	F137K + R1K PCR	Aftorf34 R1-p3r	137350	137331	R	GAGCTACTCTAAGAGGTAGG
288	F137K + R1K PCR	Aftorf34 R1-p2	137097	137115	F	CTGAGAATAGGCGCAGACC
289	F137K + R1K PCR	Aftorf34 R1-p3	137464	137483	F	GTCATGTTGCCTGCTGAATC
290	F137K + R1K PCR	Aftorf34 R1-p4	137923	137941	F	GAGAGGATTTGAACCCCTC
291	F137K + R1K PCR	Aftorf34 R1-p5	138413	138435	F	CTTAGGGAGGTATAACAAGTATG
292	F137K + R1K PCR	Aftorf34 R1-p6	138868	138887	F	GTTGGCGCTAAGTTAGTGAG
293	F137K + R1K PCR	Aftorf34 R1-p7	139402	139420	F	GCTAACTCCCAGTGATAAG
294	F137K + R1K PCR	Aftorf34 R1-p8	139978	139995	F	CTCACGCTTCCACCTAAC
295	F137K + R1K PCR	Aftorf34 R1-p9	140491	140510	F	CAAACTACCCTGAGTGCTCC
296	F137K + R1K PCR	Aftorf34 R1-p10	141056	141073	F	GTCCCCAATCTGACTTCC
297	F137K + R1K PCR	Aftorf34 R1-p11	141648	141666	F	CACTAACCGCACTATTCAG
298	F137K + R1K PCR	Aftorf34 R1-p12	213	229	F	GTAGAACAAAATGCCCC
299	F137K + R1K PCR	orf34 F1'	899	918	F	CCTGTACCTTCTTGCATTCC

On the rows numbered 68 and 69 shown in page 3, primers used for mutation detection were shown.
