# Supplementary material for: Improved Adsorption of an Enterococcus faecalis Bacteriophage ΦEF24C with a Spontaneous Point Mutation
Source: PLoS One. 2011 Oct 25;6(10):e26648. doi: 10.1371/journal.pone.0026648 (PMC3201976; doi:10.1371/journal.pone.0026648)
Supplement: Table S3 — Primers used for cloning the genes of partial orf31 . (DOC) [file pone.0026648.s007.doc]

**Table S3. Primers used for cloning the genes of partial *orf31*.**

| **Primers** | **Direction to leading sequence** | **Sequence** | **Cloned partial *gp31*** |
| --- | --- | --- | --- |
| gp31:Sac2640 | Forward | AAAGAGCTCGCAATAGCTGATATGTCGAATGAC | *gp31:2640-4150* |
| gp31:4150Hind | Reverse | AAAAAGCTTGCTGTCTTTAAGCTGTTGTAGTTC | *gp31:2640-4150* |
| gp31:Sac3680 | Forward | AAAGAGCTCACATGGAGGCTAACTGATGAAC | *gp31:3680-5200* |
| gp31:5200Hind | Reverse | AAAAAGCTTCTCACTTGGATATGGTTGCC | *gp31:3680-5200* |
